# Traffic and Pollution Modelling for Air Quality Awareness: An Experience in the City of Zaragoza

**DOI:** 10.1007/s42979-022-01105-0

**Published:** 2022-05-07

**Authors:** Sergio Ilarri, Raquel Trillo-Lado, Lorena Marrodán

**Affiliations:** grid.11205.37 0000 0001 2152 8769 I3A, University of Zaragoza, Zaragoza, Spain

**Keywords:** Data management, Sensor data, Traffic flow modelling, Pollution modelling, SUMO, VEIN, GRAL

## Abstract

Air pollution due to the presence of small particles and gases in the atmosphere is a major cause of health problems. In urban areas, where most of the population is concentrated, traffic is a major source of air pollutants (such as nitrogen oxides or $\hbox {NO}_x$ and carbon monoxide or CO). Therefore, for smart cities, carrying out an adequate traffic monitoring is a key issue, since it can help citizens to make better decisions and public administrations to define appropriate policies. Thus, citizens could use these data to make appropriate mobility decisions. In the same way, a city council can exploit the collected data for traffic management and for the establishment of suitable traffic policies throughout the city, such as restricting the traffic flow in certain areas. For this purpose, a suitable modelling approach that provides the estimated/predicted values of pollutants at each location is needed. In this paper, an approach followed to model traffic flow and air pollution dispersion in the city of Zaragoza (Spain) is described. Our goal is to estimate the air quality in different areas of the city, to raise awareness and help citizens to make better decisions; for this purpose, traffic data play an important role. In more detail, the proposal presented includes a traffic modelling approach to estimate and predict the amount of traffic at each road segment and hour, by combining historical measurements of real traffic of vehicles and the use of the SUMO traffic simulator on real city roadmaps, along with the application of a trajectory generation strategy that complements the functionalities of SUMO (for example, SUMO’s calibrators). Furthermore, a pollution modelling approach is also provided, to estimate the impact of traffic flows in terms of pollutants in the atmosphere: an R package called *Vehicular Emissions INventories (VEIN)* is used to estimate the amount of $\hbox {NO}_x$ generated by the traffic flows by taking into account the vehicular fleet composition (i.e., the types of vehicles, their size and the type of fuel they use) of the studied area. Finally, considering this estimation of $\hbox {NO}_x$, a service capable of offering maps with the prediction of the dispersion of these atmospheric pollutants in the air has been established, which uses the *Graz Lagrangian Model (GRAL)* and takes into account the meteorological conditions and morphology of the city. The results obtained in the experimental evaluation of the proposal indicate a good accuracy in the modelling of traffic flows, whereas the comparison of the prediction of air pollutants with real measurements shows a general underestimation, due to some limitations of the input data considered. In any case, the results indicate that this first approach can be used for forecasting the air pollution within the city.

## Introduction

Nowadays, digital data management is more important than ever. Modern citizens face a variety of challenges (e.g., environmental hazards and health-related issues like the spread of the COVID-19) and the availability of good-quality data can help them make better decisions. Among the existing problems, it is known that pollution is a major source of health problems [3, 12]. Moreover, there seems to be a worrying correlation between air pollution and the dissemination of respiratory infections [11, 26, 40, 59]. More specifically, it appears that more pollution entails a higher risk of a respiratory illness, so minimizing exposure to air pollution (e.g., by reducing the time invested in parking) may help to reduce the spread of these diseases. In the case of cities, traffic is one of the main causes of the release of urban pollutants into the atmosphere [39, 42, 50]. Motivated by this fact, we are interested in providing real-time information and predictions for the next 48 h related to air quality in a city, which involves several activities: (1) the deployment of a low-cost air quality sensor network to collect air quality data about the concentration of several pollutants, such as NO, $\hbox {NO}_2$, CO and $\hbox {O}_3$; (2) the modelling of traffic flows throughout the city to have estimations of the amount of traffic per road segment and hour during the day; (3) the estimation of pollution due to these traffic flows; (4) the prediction of how these pollutants are going to disperse in the atmosphere, by considering the weather forecast (mainly the wind’s speed and direction) for the next 48-h and the shape of the city (buildings that affect the dispersion of particles in the air); (5) the publication of open data; and (6) the development of mobile applications to exploit the collected data, considering both the available real-time information and the forecast of atmospheric dispersion. For all these purposes, different types of data must be collected and integrated, but among them traffic data can be highlighted due to the high impact of traffic on air pollution.

For smart cities, modelling and managing traffic flow is, in fact, a critical topic [2, 14, 51]. However, it is generally not possible to accurately monitor the flow of cars in every road segment of a city, as this would require an expensive sensor infrastructure that must be deployed and maintained. Instead, the traffic is measured only at a few key points of the city, by deploying suitable sensors, and other techniques can be applied to extrapolate the traffic measurements to other areas of the city. To do this, a traffic flow model can be defined to attempt to estimate traffic flows in the whole city that are compatible with the few real observations available. Simulation tools, fed with the traffic measurements collected by real traffic sensors, can be used to obtain the potential traffic flows.

In addition to traffic simulation, there are two other key missing pieces to consider. On the one hand, we need to estimate the emission of pollutants from vehicles traveling in the city. For this purpose, we can use tools such as VEIN [31–33], as well as a variety of input data to drive the estimations. On the other hand, we need to estimate how those pollutants are going to disseminate in the atmosphere. For this, several tools can be considered, such as the Graz Lagrangian Model (GRAL) [25].

In this work, we describe our experience with the development of a traffic flow and pollution modelling approach for the city of Zaragoza (Spain). Even though Zaragoza is currently not facing serious pollution issues, atmospheric pollution is a cause of great and growing concern around the world, due to the impact on health. Moreover, pollution due to road traffic within urban areas is a growing concern [5]. If the problem of pollution continues and the consequent health issues raise, the regulations may be more strict and new traffic policies would need to be adopted. Therefore, the definition and deployment of approaches such as the one presented in this paper are highly valuable. The city of Zaragoza has a well-equipped network of air quality monitoring stations, and therefore it is an ideal city for testing these approaches.

With a population of around 700,000 inhabitants, Zaragoza is the fifth most populous city in Spain. It is located in the northeast side of the country, on the banks of the mighty Ebro River. Zaragoza has a cool semi-arid climate; specifically, it has warm dry summers (with daily mean around $25\,^{\circ }$C and possible high temperature over $40\,^{\circ }$C), and dry cold-to-moderate winters (daily mean temperature around $8\,^{\circ }$C). It has very little rainfall throughout the year, with an annual precipitation of about 320 mm. The number of vehicles registered in the city is about 630,000 and the fleet is mainly composed by *Light Duty Vehicles (LDV)*. Another source of air pollutants that can be considered within the city is domestic heating which, in the case of Zaragoza, mainly use natural gas as fuel.

**Fig. 1:**
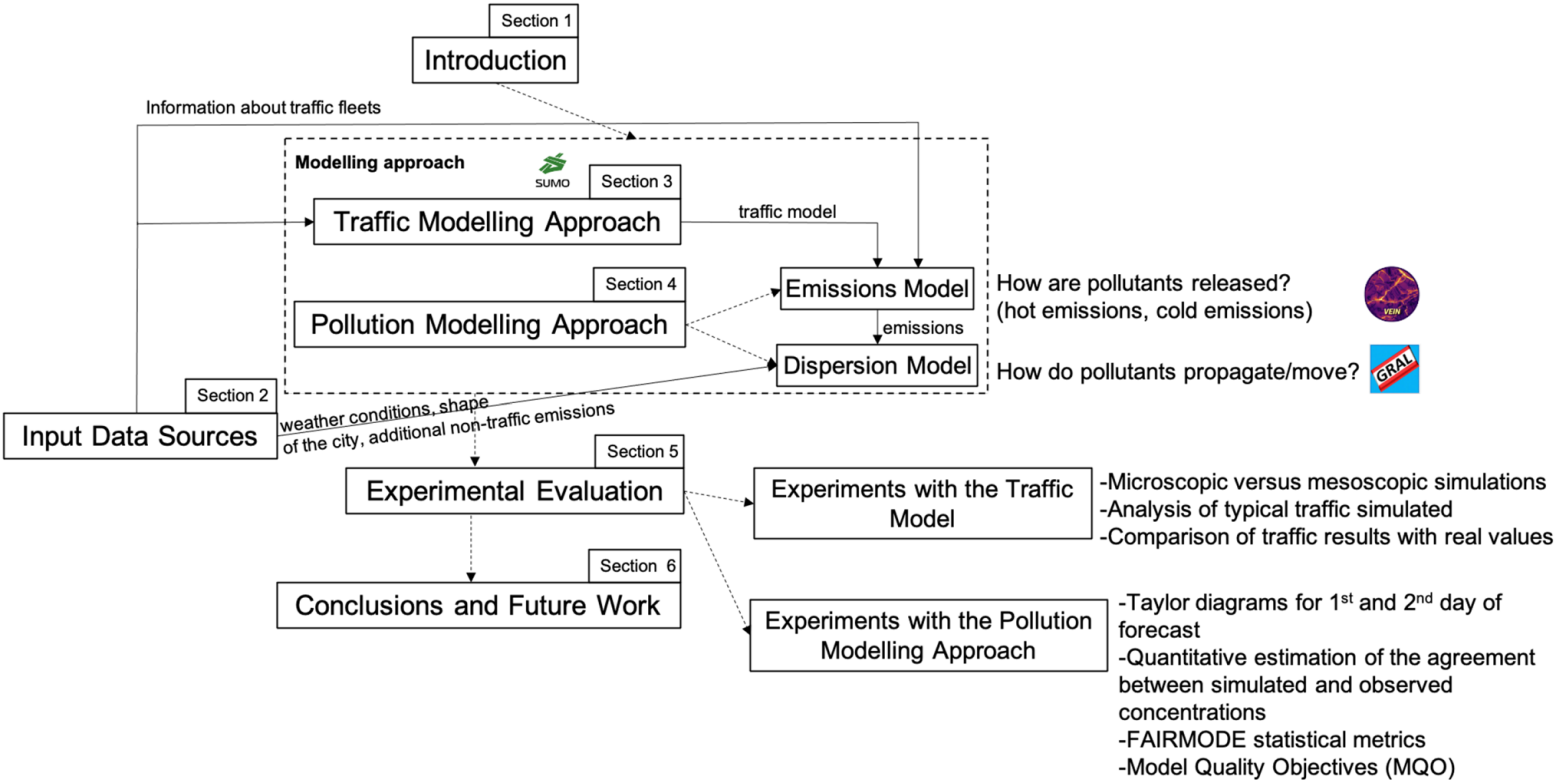
Overview of the structure of the paper

The present work significantly extends our previous conference paper [35], focused only on traffic, by including all the aspects related to pollution modelling, a description of the relevant data sources, a variety of new experiments, and more detailed and refined descriptions and explanations. The structure of the rest of this paper is as follows (see Fig. 1 for an overview). In the “Input Data Sources” section, we describe the input data sources that we consider. In the “Traffic Modelling Approach” section, we present our approach for traffic modelling. In the “Pollution Modelling Approach” section, we focus on the pollution modelling approach, i.e., how pollutant emissions due to traffic are estimated and how they are dispersed in the urban atmosphere. In the “Experimental Evaluation” section, we present the experimental evaluation that we have performed to assess the validity and benefits of our modelling approach. Finally, in the “Conclusions and Future Work” section, we present our conclusions and some future research directions.

## Input Data Sources

**Fig. 2:**
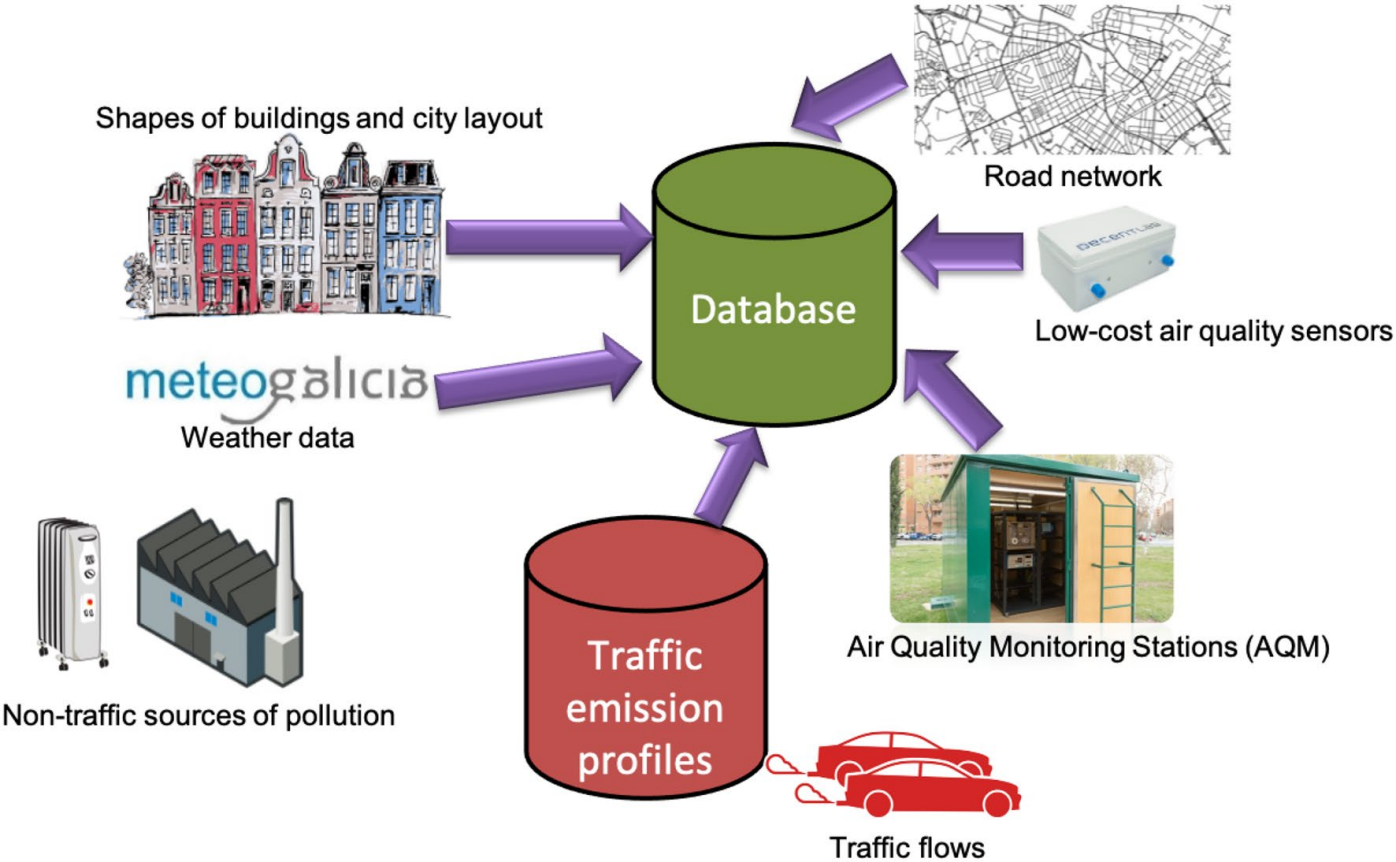
Overview of the data sources considered

In this section, we describe the different input data sources considered in this work (see Fig. 2 for an overview). First, in the “Traffic Data” section, we focus on traffic data. Road network data are explained in the “Road Network” section. Then, in the “Vehicle Fleet Composition Data” section, available data about the vehicle fleet composition for the city of Zaragoza are described. After that, in the “Meteorological Data” section, we focus on meteorological data, that play an important role in atmospheric pollutant dispersion. For the forecast of the dispersion within the urban area, it is also crucial to create a 3D representation of the city buildings, which is the focus of the “City Building Data” section. In the “Air Quality Data” section, we tackle air quality data. Finally,  the “Additional Non-traffic Emissions” section is dedicated to describing other sources of emissions produced within an urban area, beyond the traffic of vehicles. We integrate the data provided by all these data sources in a database, to facilitate their exploitation for traffic and pollution modelling; as a Database Management System (DBMS) we use PostgreSQL [54] with the PostGIS [48] extension to handle spatial data.

### Traffic Data

In this section, we describe the types of available traffic data that are collected in the city of Zaragoza. First, in the “Travel Time and Average Speed Data” section, we focus on travel time and average speed data of some city routes, which can be obtained thanks to a system based on the capture of data from Bluetooth devices. Then, in the “Traffic Counts Data” section, historical traffic data provided by other types of detectors (inductive coils and pneumatic tubes connected to traffic counters) are considered.

#### Travel Time and Average Speed Data

**Fig. 3:**
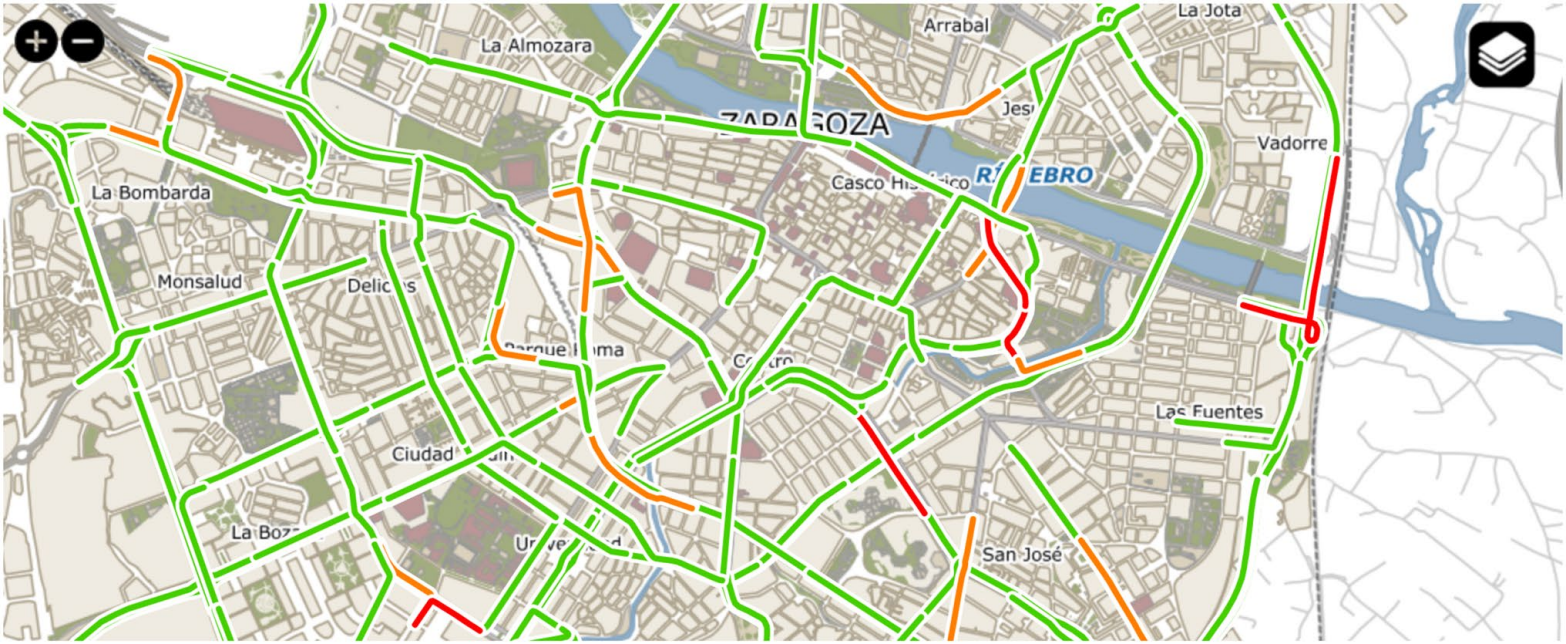
Snapshot of a portion of the real-time traffic map provided by the website of the City Council of Zaragoza (data as of March 27, 2020, at 12:40); figure extracted from [35]

**Fig. 4:**
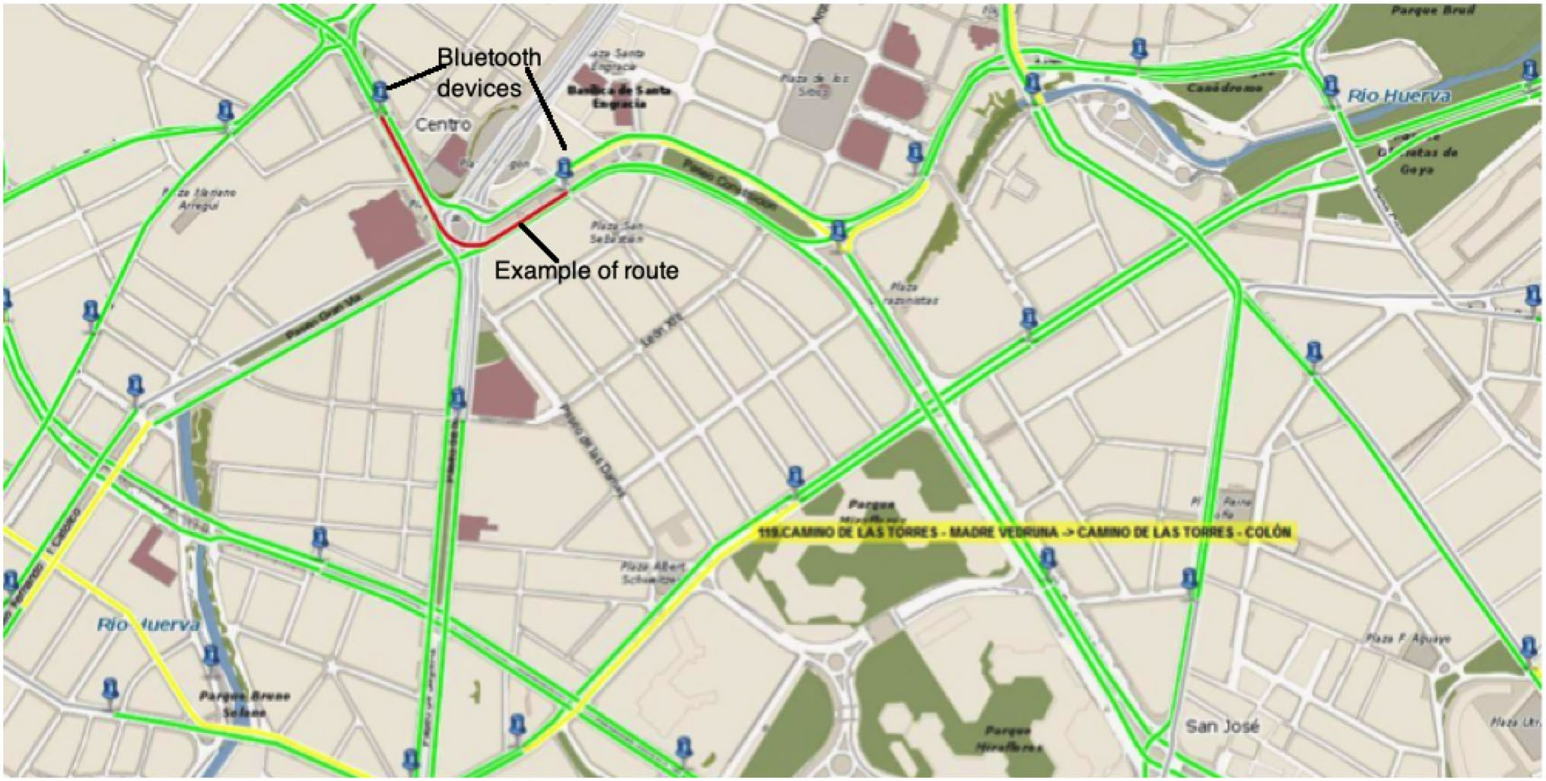
Example of a route whose average travel speed is measured using Bluetooth devices (City Council of Zaragoza); figure extracted from [35]

The Zaragoza City Council provides a map with traffic information (see Fig. 3) for some road segments (https://www.zaragoza.es/ciudad/viapublica/movilidad/trafico/trafico.htm), distinguishing among fluid, dense, and congested traffic. It is built considering the measurements obtained by several Bluetooth antennas distributed throughout the city, which use Worldsensing’s Bitcarrier Traffic Flow Management technology [57, 58]. Furthermore, specific routes from one antenna to another antenna, denoted as “links”, are also defined (see Fig. 4 for an example). The average speed of the vehicles that went through a link within a specific time interval (5 min) is computed by considering the distance between the antennas and the time needed by the vehicles to traverse that link. Moreover, real-time traffic information containing the travel time of certain routes (origin-destination, usually represented as an intersection of two roads and/or popular points of interest in the city) is published as open data (at https://www.zaragoza.es/sede/portal/datos-abiertos/servicio/catalogo/291) and in JSON format (http://www.zaragoza.es/trafico/estado/tiempos.json).

With these data, it would be possible to define a partially-filled origin-destination matrix with several travel times, which is insufficient for the purposes of this work since, as stated before, only some routes of the city are covered (for example, a query submitted on March 25, 2020, returned only 24 routes). Moreover, only data about travel time are available and we need specific data of the number of vehicles in as many road segments of the city as possible.

As a potential alternative data source, *Google Maps* [24] offers an overall view of the traffic density in different areas of a city (a green color is used to represent no traffic delays, orange is used for a medium amount of traffic, and red indicates traffic delays—the darker the red, the slower the traffic—) as well as information related to several types of traffic incidents (accidents, constructions, road closures, and other incidents). Besides, there is an option to visualize either the live traffic or the typical (expected) traffic. It covers many streets in the city of Zaragoza (although some secondary streets are not currently considered, according to what we have observed on March 11, 2020). Besides, it does not provide fine-grained traffic information such as the counts of vehicles on different road segments.

As we need more fine-grained data, covering as much of the city as possible, instead of using these travel time and average speed data we will use historical traffic counts data provided by static traffic sensors, as described in the “Traffic Counts Data” section; thus, with the historical traffic counts data we have precise traffic counts for 46 road segments. Nevertheless, the types of data described in this section could potentially be used to feed our SUMO traffic model (described in the “Traffic Modelling Approach” section) with real-time travel data, to refine it. However, including these data is not direct and an in-depth analysis of the required strategy would be required, since we need data about the number of vehicles on the road segments as an input to SUMO.

#### Traffic Counts Data

**Fig. 5:**
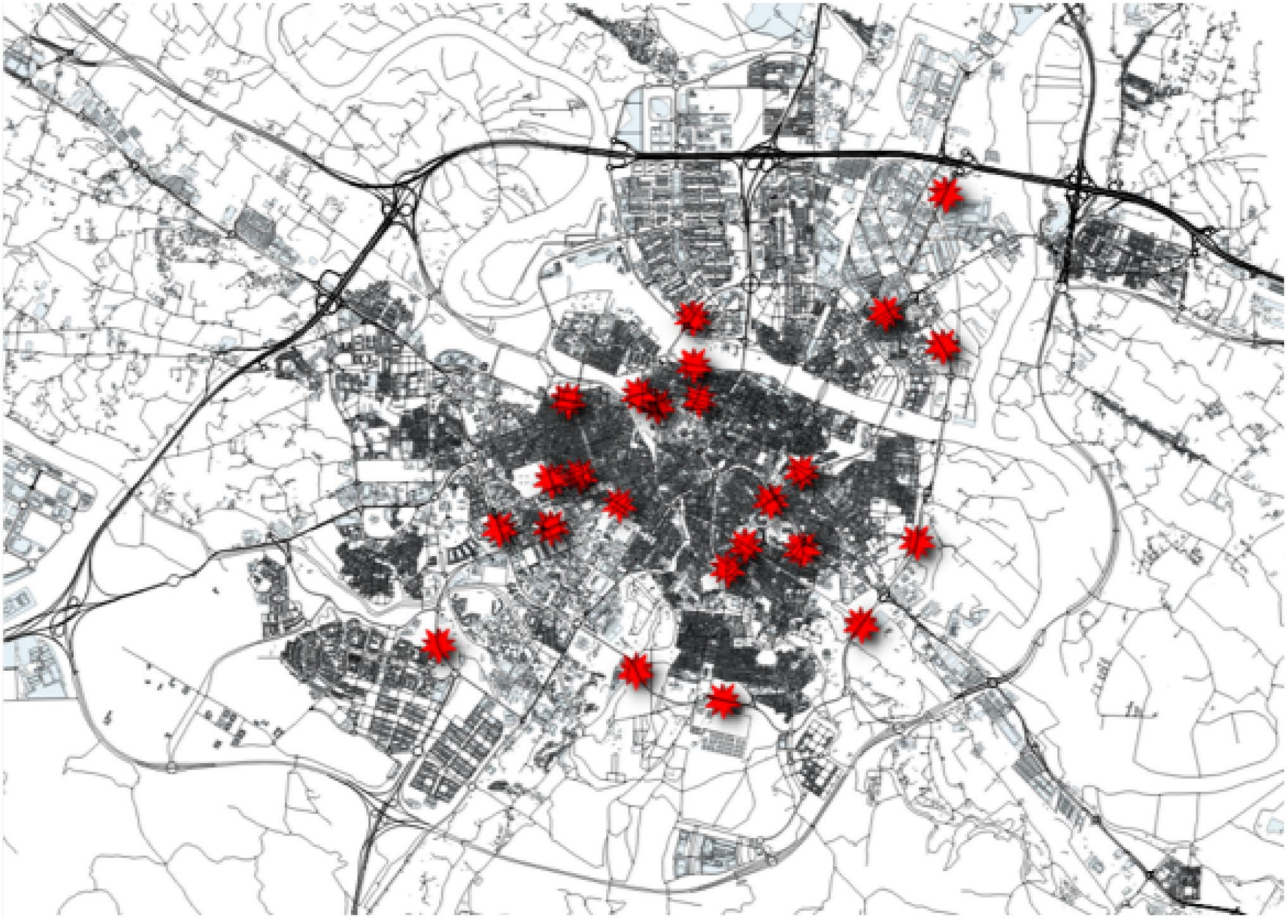
Static traffic devices in the city of Zaragoza (snapshot of QGIS); figure extracted from [35]

**Fig. 6:**
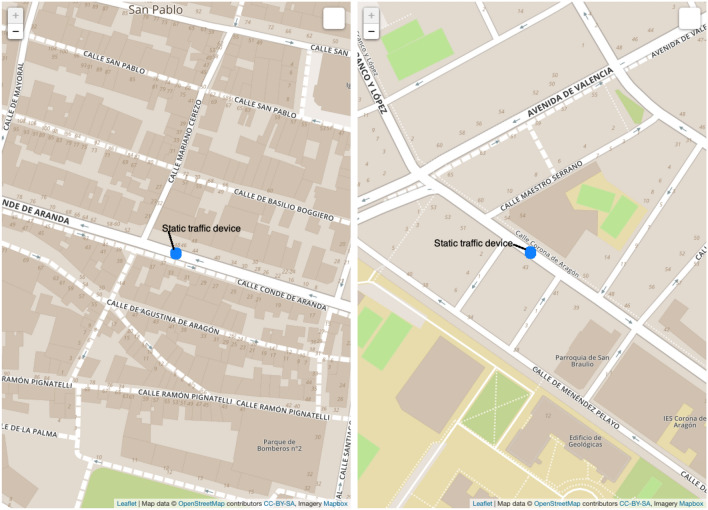
Example of two static devices measuring traffic on two road segments in just one direction (maps provided by OpenStreetMap; screenshots of the spatial data viewer of the DBeaver tool, available at https://dbeaver.io); figure extracted from [35]

Fortunately, the Zaragoza Traffic Control Center also provided us with some historical data obtained from both static traffic devices and mobile traffic devices that measure the traffic flow in the different road segments of the city:

- Static traffic devices (called “permanent stations”) are 46 devices installed in different positions of the city of Zaragoza, as shown in Fig. 5, generated using QGIS [49], where the static traffic devices are highlighted in red. These devices are inductive coils located under the asphalt that provide traffic data 24 h a day for every day of the year, which is why they are said to be “permanent”. Usually, there are two devices in the same road, one for each direction of circulation. However, there are two road segments where there is only one device measuring the traffic in just one direction (see Fig. 6).
- Mobile traffic devices (termed “annual stations”) are mobile devices installed in different points of the city along the year (e.g., in a total of 546 different locations in 2019 and 652 in 2020). More specifically, they are pneumatic tubes on the roads connected to traffic counter devices. As in the case of static devices, there are usually two devices on the same road (one for each direction of circulation), but there are also exceptions. There is a set of predefined locations where these mobile devices can be located; however, in each location there is a device measuring traffic only for a few days (e.g., in 2019 an average of 3 days with a standard deviation of 0.63), as the static devices are moved around these defined locations from time to time. The predefined locations are called “annual stations” because the devices installed there try to predict the average annual traffic density in work days or “Intensidad Media Laborable” (IML) in Spanish.

Using these historical traffic data, we can feed SUMO with the information needed to build our traffic model, which is able to estimate the traffic flow for each road segment of the city at any time instant, as we describe in the “Traffic Modelling Approach” section.

### Road Network

The *roadmap* of the city of Zaragoza, obtained from *OpenStreetMap* [47] in OSM (OpenStreetMap) format, allows to retrieve the different road segments where traffic flows should be considered as a source of pollution emissions. It includes, besides the road graph, information such as the number of lanes and speed limit of each road section, turn restrictions, and the presence of traffic lights. The roadmap is stored in our database to facilitate its use.

**Fig. 7:**
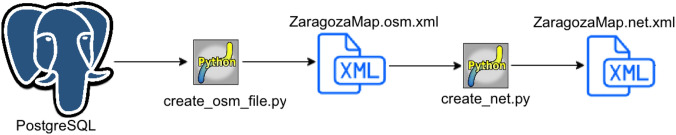
Workflow used to create a roadmap in the format required by SUMO; figure extracted from [35]

In Fig. 7, we show the workflow defined for the creation of a roadmap in the format required by SUMO. First, a Python script queries the database, to obtain information about the roadmap of Zaragoza, and generates a file with the roadmap in OSM format. Then, another Python script takes the OSM file and transforms it into a roadmap file compatible with SUMO, by using the SUMO tool *netconvert* [21].

### Vehicle Fleet Composition Data

To estimate the emissions of pollutants in the city of Zaragoza, not only information about the traffic flows is needed, but also data about the types of vehicles, the type of fuel they use, their year of registration, number of cylinders, and maximum load. This information is what we call *vehicle fleet composition*, and has been obtained from the General Direction of Traffic of the Spanish Government. These data have been directly downloaded from the official website (https://sedeapl.dgt.gob.es/WEB_IEST_CONSULTA/categoria.faces) and stored in the database. For this purpose, a suitable *Extract, Transform and Load (ETL)* process has been defined to load the data in the database. As the download of data from the website is protected by a captcha, the downloading step has to be performed manually.

### Meteorological Data

To perform the dispersion simulations, information about weather forecasts is required. The data used in this work have been provided by MeteoGalicia, which is the Galician Regional Weather Service (DX de Calidade Ambiental e Cambio Climático, Xunta de Galicia). The *Weather Research Forecast (WRF)* from MeteoGalicia provides hourly data with a 12 $\times$ 12 $\hbox {km}^2$ grid resolution for the city of Zaragoza. This meteorological information mainly includes wind data (speed and direction), vertical temperature variation, and solar radiation data, to compute the *Stability Class (SC)* according to the US-EPA SRDT method [16]. The stability class represents a simple way to classify the atmospheric conditions that are critical for the dispersion of pollutants.

### City Building Data

A 3D representation of the city buildings is needed for a correct estimation of the dispersion of pollutants in the atmosphere. This information has been obtained from the Spanish cadastre (http://www.catastro.minhap.es), where a database of urban cadastre is available, from which geometric representations of buildings of urban areas can be obtained. Each building, among other attributes, is characterized by a geometric polygon that defines its shape and a textual attribute that encodes the type of construction and the number of floors. The data model of the dataset corresponds with the INSPIRE Directive for the spatial theme *Buildings* [36]. The downloaded dataset contains two features types: *Building* and *BuildingPart*. In *BuildingPart*, there is an attribute that is particularly relevant to our purposes: *numberOfFloorsAboveGround*, which contains the number of floors above ground, from which the height of the building can be estimated by considering an approximate height of 3 meters for each floor. In this way, it is possible to construct a 3D representation of each building. Once the height has been estimated, shapefiles containing this information are provided to GRAL as input data.

### Air Quality Data

In this work, two sources of air quality data can be distinguished: *Air Quality Monitoring (AQM) stations* (described in the “Air Quality Monitoring Stations (AQM)” section) and low-cost sensors (described in the “Low-Cost Sensors” section).

#### Air Quality Monitoring Stations (AQM)

The Zaragoza City Council has installed 8 air quality regulatory stations throughout the town, well equipped to monitor the concentrations of several air pollutants in representative locations of the city with different traffic characteristics. The names/locations, official identifiers, and site types of the AQM stations (the site type depends on the environment where the station is located, which can be an industrial zone, an urban traffic zone —close to urban roads with traffic activity—, or a background zone) are reported in Table 1. The main purposes of the data provided by these stations are to calibrate low-cost air quality sensors (as briefly described in the “Low-Cost Sensors” section), to create real-time urban air-quality maps, and to test and validate pollution dispersion forecasts obtained.

Data about the concentration of several atmospheric pollutants (e.g., $\hbox {NO}_2$, CO, particulate matter, and $\hbox {O}_3$) are published every hour at the city council’s official website (https://www.zaragoza.es/sede/portal/medioambiente/calidad-aire/). We obtain those data from the Open Data Portal of the city council (by querying a SPARQL endpoint, available at https://www.zaragoza.es/sede/portal/datos-abiertos/servicio/sparql); besides, data obtained with a higher frequency are provided to us by email for the calibration of the low-cost air quality sensors described in the “Low-Cost Sensors” section.

**Table 1:** Names/locations, identifiers, and site type for air quality monitoring (AQM) stations in the city of Zaragoza

Name/location	Identifier	Site type
Actur	40	Urban background
Avda. de Soria	39	Urban traffic (intense traffic)
Centro	38	Urban traffic (moderate traffic)
El Picarral	26	Urban traffic (moderate traffic)
Jaime Ferrán	32	Suburban traffic (moderate traffic)
Las Fuentes	37	Urban traffic (intense traffic)
Renovales	36	Urban background
Roger de Flor	29	Urban traffic (intense traffic)

#### Low-Cost Sensors

We have deployed 10 low-cost air quality sensors (manufactured by Decentlab GmbH, Duebendorf, Switzerland) across the city of Zaragoza, covering areas with different urban traffic conditions (intense or moderate traffic). Each unit consists of several Alphasense electrochemical cells to estimate NO, $\hbox {NO}_2$, CO and $\hbox {O}_3$ pollutants, along with the relative humidity and temperature. The units are calibrated by co-location at the AQM stations and subsequently installed at a specific location of the city. For each sensor, the raw readings (i.e., the mV measured by the electrochemical cells) are collected, and a regression model using a random forest [7] is applied to obtain a calibration function and translate the raw data into concentration values, exploiting the data from the regulatory stations during co-location periods.

### Additional Non-traffic Emissions

In an urban environment, in addition to road traffic, there are other sources of pollutant emissions that must be taken into account for a good forecast of pollutant dispersion within an urban area. In our work, we have also considered the following ones:

- *Domestic heating emissions*. During winter time, emissions from domestic heating within the city are not negligible. Therefore, they have also been considered in our pollution modelling approach. The $\hbox {NO}_x$ annual total (ton/year), estimated by the Environmental Agency of Zaragoza Council for the year 2015 [4], has been distributed throughout the city of Zaragoza taking into account the distribution of population in the different neighborhoods of the city. Moreover, the external air temperature is also considered to estimate the need of using domestic heating. So, by forecasting the external air temperature, it is possible to have an estimate of the expected non-industrial combustion emissions for the following days.
- *Waste management emissions*. Within the spatial domain considered for the city of Zaragoza, there are some waste management plants whose emissions have also been considered as input data for our pollution modelling approach. Specifically, these plants are: the municipality cemetery located in the “Torrero” neighborhood and a *Wastewater Treatment Plant (WWTP)* located in the “La Almozara” neighborhood. The same source described above [4] has been used to estimate emissions from waste management treatments.
- *Main industrial combustion emissions*. $\hbox {NO}_x$ emissions from the three main industrial activities within the spatial domain considered for the city of Zaragoza have been used, taking into account the height of their chimneys, where pollutants are emitted. They operate 24 h a day with an almost constant rhythm, which has been reflected in the estimation of pollutant emissions.

All these additional data sources are considered in our pollution modelling approach, by using them as input to the software GRAL [25], which is used to estimate the dispersion of pollutants (for more details, see the “Graz Lagrangian Model (GRAL)” section).

## Traffic Modelling Approach

As explained in the “Input Data Source” section, we have historical real traffic data measurements for some road segments in the city, but we need to estimate the traffic at a certain time instant in all the road segments. For this purpose, we need to develop a traffic model.

**Fig. 8:**
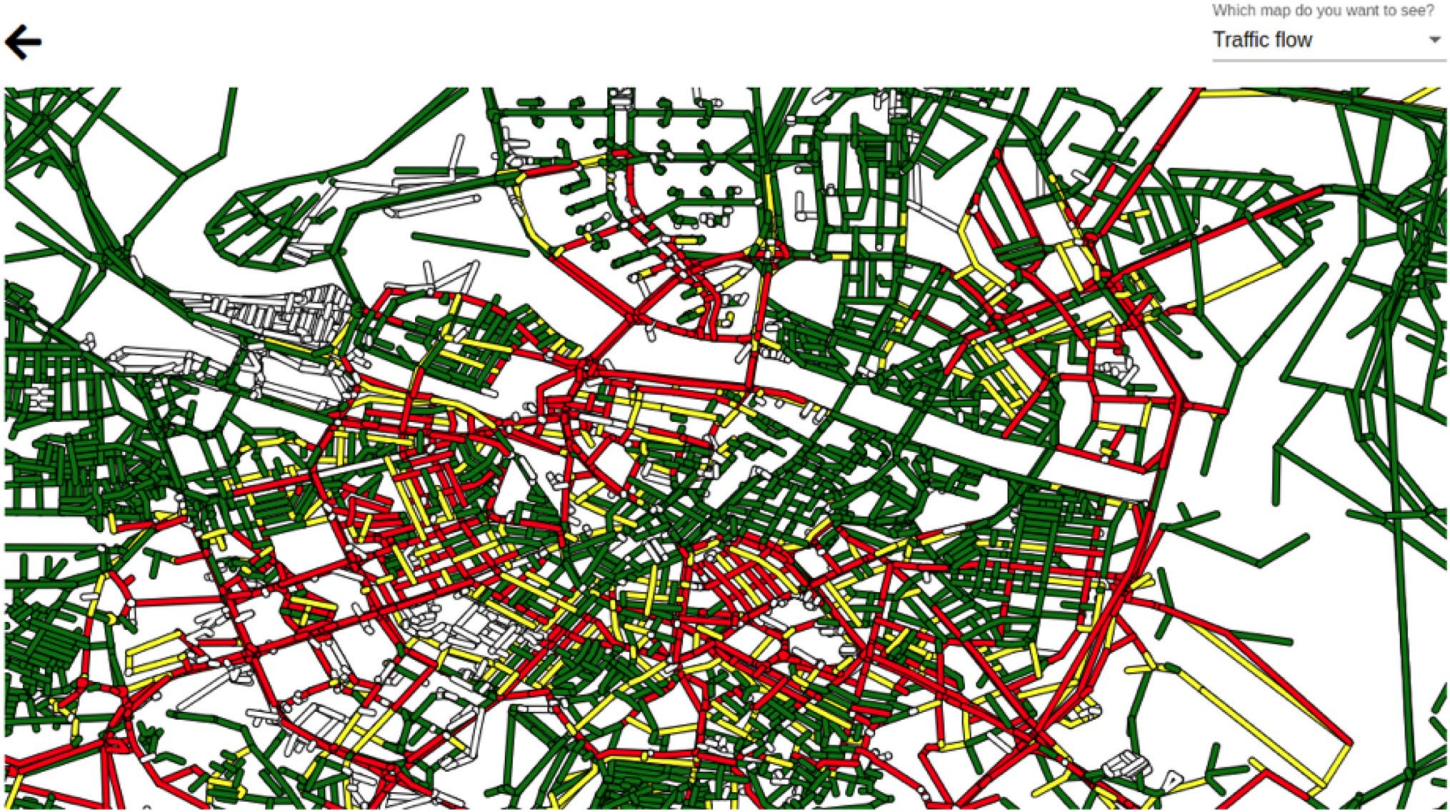
Traffic flow simulation for an expected special event; figure extracted from [35]

**Fig. 9:**
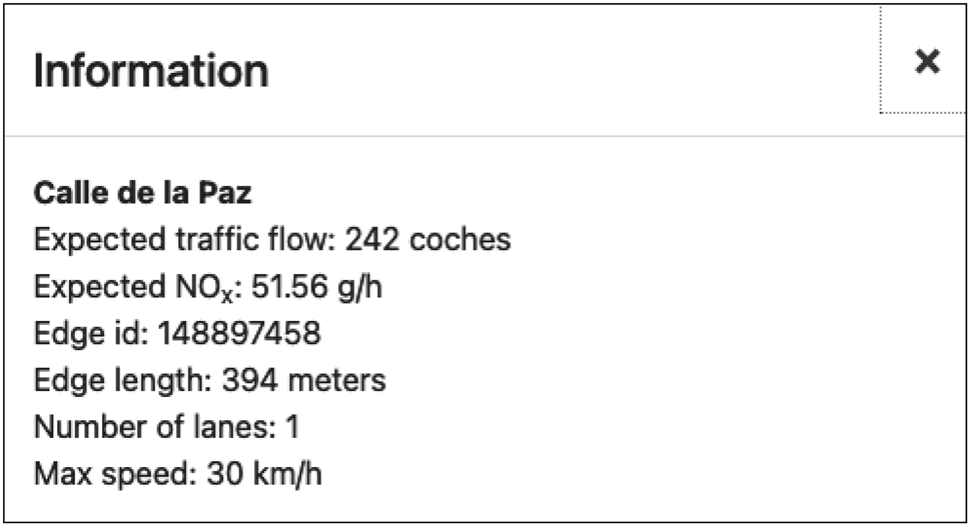
Data shown for a position clicked on the map of the GUI

For an easy exploitation and testing of the traffic model by end users, we have developed a Graphical User Interface (GUI), which supports basic interaction and visualization of the traffic flows in a user-friendly way. The user selects the input data and can also indicate optional information in case there is some special event in the city that can affect the expected traffic flows. As an example, in Fig. 8, we show a snapshot of the traffic flow map computed for a day with a special event that implies traffic higher than usual. The map is interactive, and so, for example, the user can move around the map, zoom in or out, or click on a specific location to obtain details (an example is shown in Fig. 9).

In this section, first we provide an overview of the traffic modelling approach (“Overview of the Traffic Modelling Approach” section), and then we describe the components developed (“Components Developed for Traffic Modelling” section).

### Overview of the Traffic Modelling Approach

A possible approach to obtain a traffic model is to use a traffic simulator. For example, VanetMobiSim [28, 29] is a Java-based simulator focused on *vehicular ad-hoc networks (VANETS)* [34], and MAVSIM [56] is a simulator specifically designed to test applications for VANETS that are based on the use of mobile agent technology [55] for distributed data management. With these types of simulators, that offer functionalities for vehicle mobility simulation, different types of vehicle mobility models [8, 27] could be applied, such as the *Random Waypoint Model (RWM)*, the *Graph-Based Mobility Model (GBMM)*, the *Constant Speed Motion (CSM)* model, and the *Smooth Motion Model (SMM)*. These mobility models allow a simulation of traffic at the individual vehicle level (microscopic simulations), but unfortunately they do not support the combination of those mobility models with real input traffic data, which is needed in our case to exploit the real traffic data available (see the “Traffic Counts Data” section).

We are rather interested in obtaining realistic simulations that are consistent with real traffic observations. To achieve this goal, it is essential to be able to feed real traffic data as input for a traffic simulation. The *Simulation of Urban MObility (SUMO)* simulator is a popular simulation tool, which supports the definition of *calibrators* [23] to regulate the traffic in specific segments according to the expected traffic values. There are also simulators that consider communication network aspects, such as *Vehicles in Network Simulator (VEINS)* [52, 53], which is an open source software that supports the re-routing of vehicles based on network messages received, and it is based on SUMO for the simulation of traffic and OMNeT++ [46] for the simulation of network communications. However, in our case, we are interested in the mobility of vehicles and simulating network communication aspects is not among our needs. Therefore, in this work, we use and evaluate SUMO, considering both microscopic and mesoscopic simulations and complementing SUMO’s built-in capabilities (such as the use of calibrators) with other simulation strategies for traffic regulation. Regarding the type of traffic flow model considered, it should be noted that SUMO provides two of the three (only macroscopic models are not supported) main types of traffic flow models identified in the literature [38]:

- *Microscopic simulations* [10, 41], where the dynamics of each vehicle are modeled individually. This is the default simulation model of SUMO.
- *Mesoscopic simulations* [15]. A mesoscopic model combines features of microscopic simulations and macroscopic simulations (that focus on average vehicle dynamics like the traffic density). Specifically, the mesoscopic model of SUMO, which is based on the work presented in [15], “computes vehicle movements with queues and runs up to 100 times faster than the microscopic model of SUMO” [19].

We have evaluated and compared both types of models in our experiments (see the “Microscopic Versus Mesoscopic Simulations” section). We concluded that a mesoscopic model minimizes the number of errors, and therefore we integrated that type of model in our prototype.

**Fig. 10:**
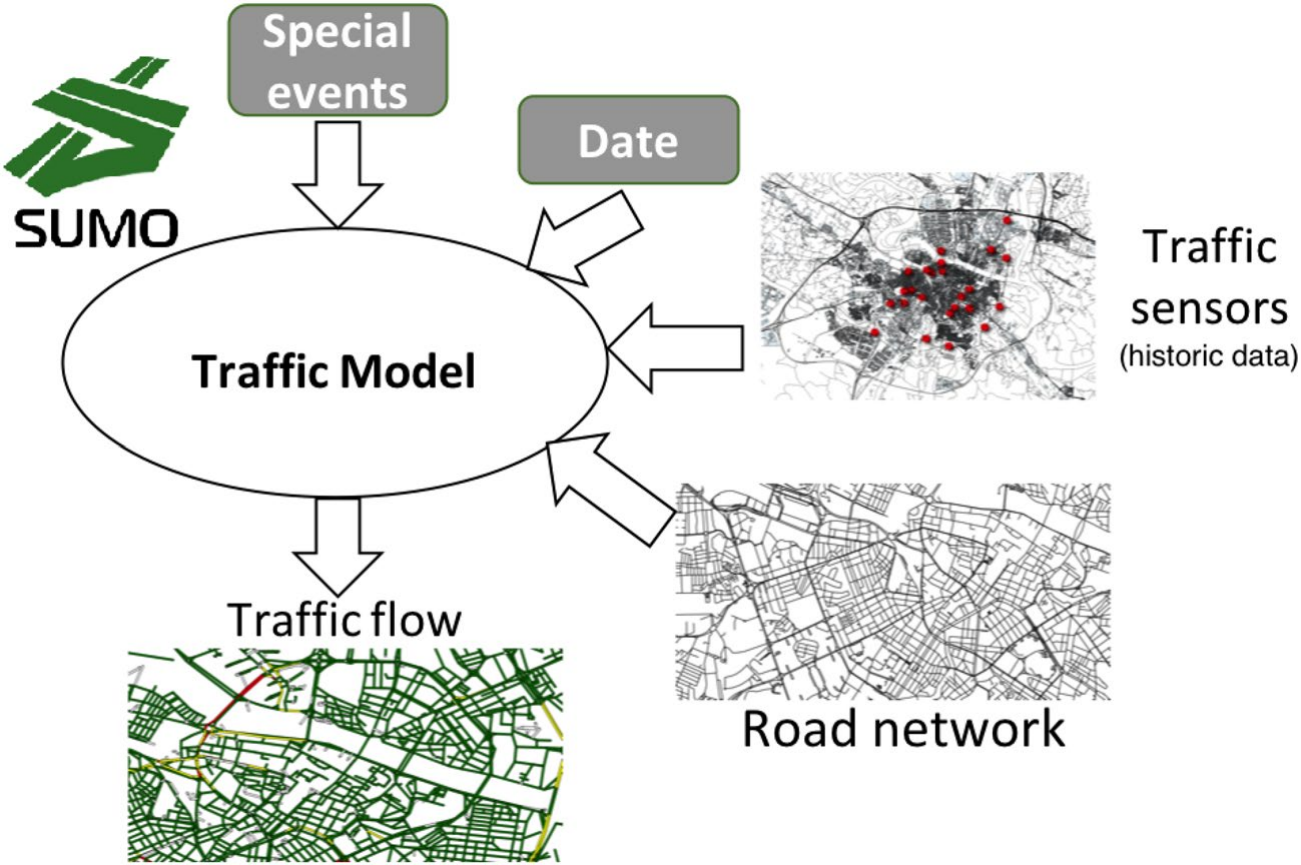
Overview of the traffic modelling approach; figure extracted from [35]

In Fig. 10, an overview of the traffic modelling approach proposed is shown. The goal of the traffic model is to estimate traffic data on each road segment of the city (i.e., the number of vehicles passing through that road segment during each hour of the day and their average speed) based on a limited set of observed data. More specifically, we exploit historical real traffic observations that are available only for some road segments (the segments where there is a static traffic device, as explained in the “Traffic Counts Data” section). In this way, we can obtain an overall picture of the traffic in any part of the city without the need to install sensors along all the road segments, which would be very expensive. Instead, we only exploit the data captured by the already-existing sensors installed in the city. Two parameters are considered as an input to the proposed traffic modelling approach:

1. A *date* parameter, which represents the date for which an estimation of the traffic flows throughout the city is required. It could be a past day (past traffic data estimation) or a future date (traffic prediction). When the input is a past day for which real traffic observations are available, the traffic model will estimate the traffic in all the road segments of the city based on the available real observations. If the input is a future date, then the traffic model will try to predict the traffic in the city during that day based on the historical data available for some of the city road segments.
2. Information about *special events*, which could require fine-tuning some parameters of the generated models. For example, strict forced transportation constraints (e.g., due to environmental protection or emergencies like the one caused by the COVID-19) can impose strong limitations on the existing traffic in a city. This situation could be temporary, only produced due to some special event. For example, traffic was considerably reduced at the beginning of the COVID-19 pandemics (e.g., according to TomTom’s data, Madrid’s traffic decreased by $96\%$ during the first weeks of the state of alarm/emergency decreed in March 2020 in Spain due to the COVID-19 [13]), but this situation changed later as the constraints started to be relaxed; indeed, with a spreading disease like the COVID-19, existing traffic may aggravate due to the potential preference for single-occupancy vehicles as opposed to public transportation [30]. Overall, as these are unexpected situations, the impact of these events may lead to traffic following trends quite different from the ones observed in the past. Therefore, this input to the traffic model is used to adjust the models based on this information, for example, by automatically reducing the expected traffic in Zaragoza by a certain percentage.

**Fig. 11:**
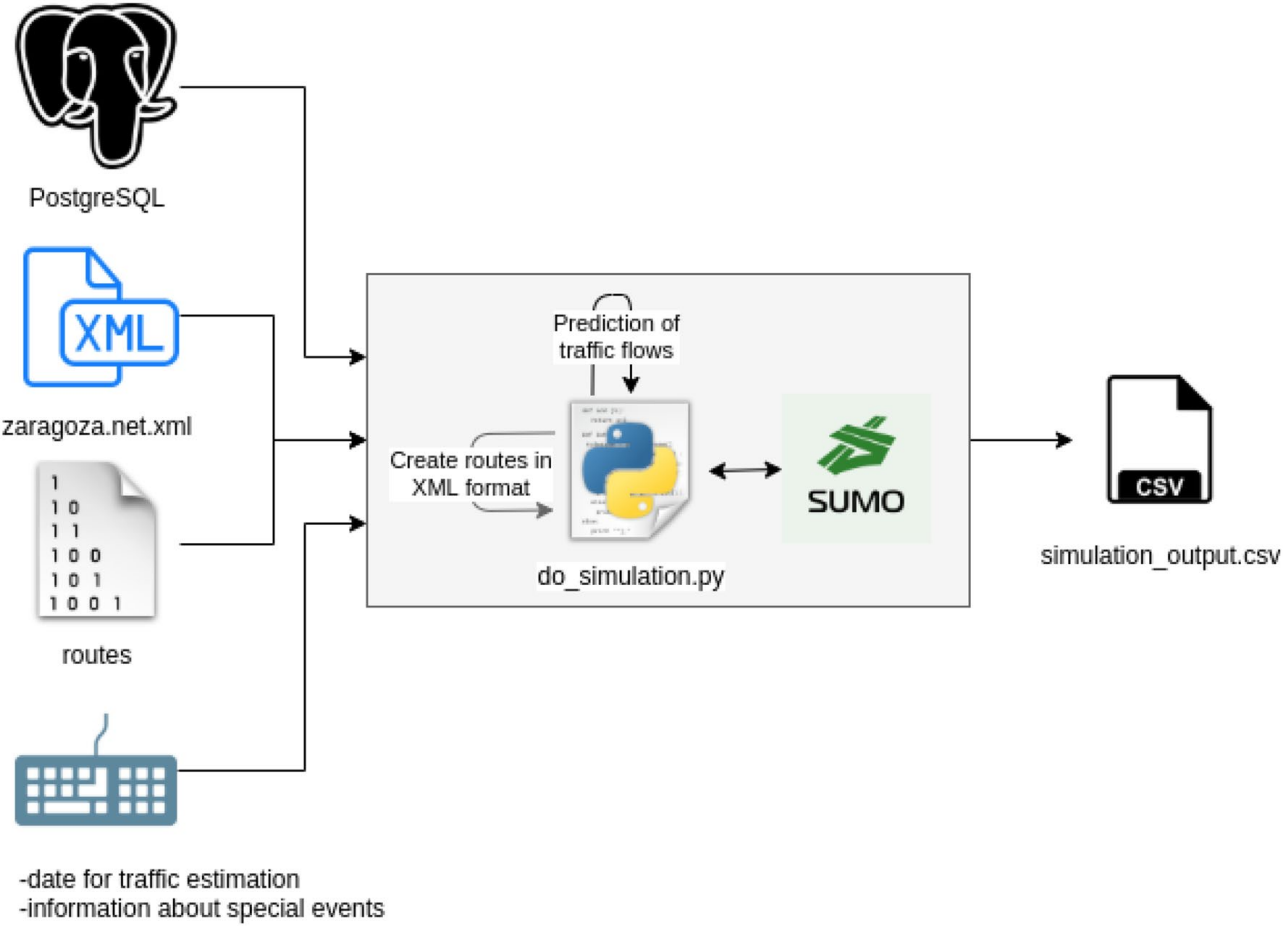
Workflow used to estimate traffic for a given date; figure extracted from [35]

The workflow defined for the generation of traffic data for a given date is shown in Fig. 11. A Python script handles the input parameters described above, interacts with SUMO, and retrieves SUMO results in CSV format, which contains a row for each road segment and hour during the day. Each row includes different fields such as the edge identifier (a road segment/edge is a street or a part of a street, as defined by the edges in OSM), the hour of the day, the number of vehicles passing through that segment at that hour, and their average speed.

### Components Developed for Traffic Modelling

**Fig. 12:**
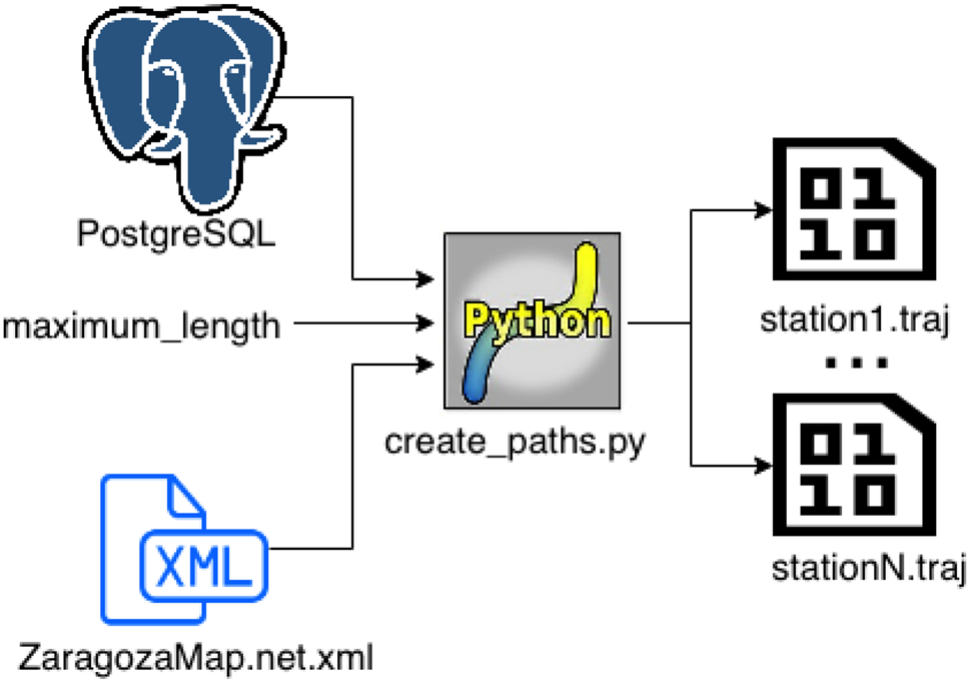
Workflow followed to generate possible routes to be used by vehicles in SUMO; figure extracted from [35]

Our traffic modelling approach is based on the use of a traffic predictor, a route generator, a route allocator, and SUMO’s calibrators. More specifically, for the simulation of traffic with SUMO, three components have been defined and implemented:

1. A *traffic predictor*, whose goal is to predict the expected traffic flow that will be measured by the traffic stations on a (future) date for which no actual data are (yet) available. For this purpose, a multiple linear regression [1] is applied on the real historical observations provided by the traffic stations for all the dates in our historical dataset. As predictors, we use the id of the traffic station, the real traffic data observed by that traffic station, and the month, hour, and type of day (weekday, Saturday, or holiday) for that observation.
2. A *route generator*, which computes routes that can be used by the vehicles within the SUMO simulation (see Fig. 12). It should be noticed that the historical routes actually followed by the vehicles are not available input data, as we only have information about the traffic flows at specific locations in the city. The strategy used for the generation of routes is as follows:
  For each traffic monitoring device, all the possible routes passing through the road segment attached to that device (which we call the *target road segment*) are computed. A maximum route length is considered (in our prototype, 30 edges), to avoid lengthy computations, such that only the routes passing through the considered road segment and smaller than the maximum route length are actually computed.
  Besides, the *route latency*, which is the minimum amount of time needed to reach the target road segment by following that route, is computed. This minimum latency can be estimated by considering that the car moves through each road segment at its maximum allowed speed and that all the traffic lights along the route are green.
  The output of this process is, for each traffic monitoring station, a list of possible eligible routes passing by that station.
3. A *route allocator* per traffic monitoring station, which randomly assigns routes pre-calculated by the route generator to vehicles during a simulation with SUMO. The assignment of routes should be compatible with the traffic observations at each traffic monitoring station. For example, if during the hour of the day that is being simulated at the moment there are 200 vehicles that should pass by traffic monitoring station EP2.1, then we have to generate 200 vehicles and assign to each of them a route that passes by that station (randomly selected among the pre-computed routes for that station). In our current prototype, all the pre-computed routes whose route latency is smaller than 1 h are eligible, but the probability that a specific route is selected increases with the number of road segments it contains (to minimize the number of short routes generated) and with the presence of major city roads such as avenues or main roads along the city (as routes traversing those popular roads are more likely). Furthermore, the route allocator tries to distribute the passage of vehicles by each station as uniformly as possible during the hour that is being simulated. The motivation for doing this is that it is usually more realistic than having large peaks of traffic at specific moments within the hour. For this purpose, for each traffic monitoring station, each hour is divided into 3600/*numVehicles* intervals (seconds per vehicle), where *numVehicles* is the total flow of vehicles expected to be detected by that station during that hour; then, during each of those intervals one vehicle is scheduled to pass by that station (the moment when each vehicle should start its trajectory is estimated based on the origin of its route and the time when it is scheduled to pass by the traffic monitoring station).

Besides, the use of the previous components are combined with the use of SUMO *calibrators* [23], which are devices that try to regulate the amount of traffic passing through the edge where they are located according to the expected traffic flow specified for that calibrator (through an input XML file). In our case, we attach one calibrator to each edge where a static traffic monitoring station is located; then, we assign to the calibrator a target traffic flow equals to the expected traffic flow on that road segment (i.e., the real traffic observation, if available, or otherwise, the predicted traffic flow). SUMO calibrators apply an algorithm, described in Ref. [17], to insert or remove vehicles, as needed, when it is expected that the target traffic flow will not be reached. We have decided to use random routes for the additional vehicles that may be inserted by SUMO, although SUMO also supports assigning fixed routes.

The use of calibrators represents a complementary strategy to the use of our defined route allocator. Thus, notice that the route allocator operates under uncertainty, which may lead to sub-optimal results. On the one hand, as route allocators act independently for each traffic station, the impact of the allocations performed by one route allocator are not considered by the other route allocators when performing their allocations: as a route passing by one station may also pass by other stations, this may lead to an increased number of vehicles for some stations. On the other hand, the real route latency can actually be larger than the one estimated (e.g., due to traffic jams), which could decrease the final number of vehicles passing by a given station. These effects can be minimized thanks to the use of calibrators. Although it is possible to use only calibrators and the *randomTrips.py* script of SUMO to generate routes randomly, the use of our own route trajectory generator and route allocators gives us more control over the final trajectories followed by the individual vehicles.

## Pollution Modelling Approach

In this section, we discuss the pollution modelling approach followed. First, in the “Emissions Model” section, we describe the emissions model, and then, in the “Dispersion Model” section, we describe the dispersion model.

### Emissions Model

**Fig. 13:**
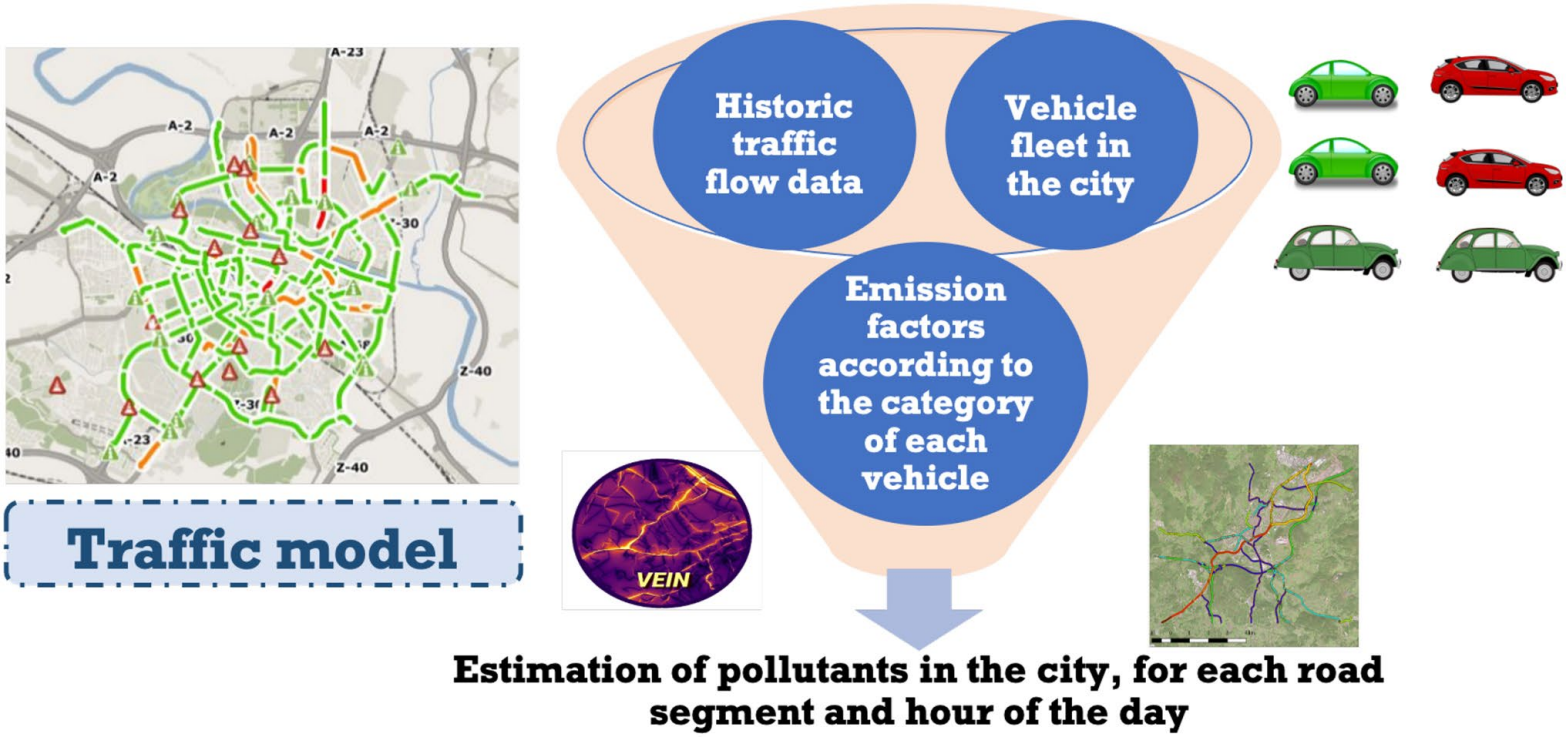
Use of VEIN to compute the emissions

The goal of the emissions model is to compute the amount of $\hbox {NO}_x$ emissions ($\hbox {NO}_2$ and NO) per distance traveled (kg/km) produced by the traffic flow on each of the street segments of the city (see Fig. 13). So, we map traffic flows into the emission of pollutants. Two types of emissions are considered: hot and cold emissions. The former occurs when the vehicle’s engine is at its normal operating temperature, while the latter occurs in the engine’s starting phase.

To achieve this objective, we have used *VEIN* [31–33], which applies hot and cold emission factors based on the Emission Inventory Guidebook by the *European Environment Agency (EEA)*  [43]. It is an R package that allows estimating the emissions produced by a vehicle based on the type of vehicle, the speed at which it circulates, and the ambient temperature.

More specifically, we have developed a *calculate_emissions.R* script, that uses the VEIN library. The script mainly uses the following functions defined in VEIN:

- *ef_hdv_speed*, which computes the emission factors for *Heavy Duty Vehicles (HDV)* based on the average speed.
- *ef_ldv_speed*, which computes the emission factors for *Light Duty Vehicles (LDV)* and motorcycles based on the average speed.
- *ef_ldv_cold*, which computes the cold start emissions for LDV.

The core algorithm that computes the emissions for a given flow of vehicles is shown in Algorithm 1. The overall process is as follows. First, we read a CSV file that contains the information about the traffic fleet (types of vehicles and their proportions). Then, we read from our database the predicted traffic flows in each segment of the city for the data desired. For each segment, we calculate the pollutants released by calling the previous *calculate_emissions* function (Algorithm 1). Finally, we store the predictions of pollutants in the database and, if needed, also in an output CSV file for further processing. A number of parameters allow configuring the required behavior of the main R script; for example, we can compute the pollutants only for the rush hour (i.e., the hour with the highest number of vehicles) or for each hour of the day. As we do not compute the emissions in real-time, we estimate the ambient temperature based on average historical values for the corresponding month.

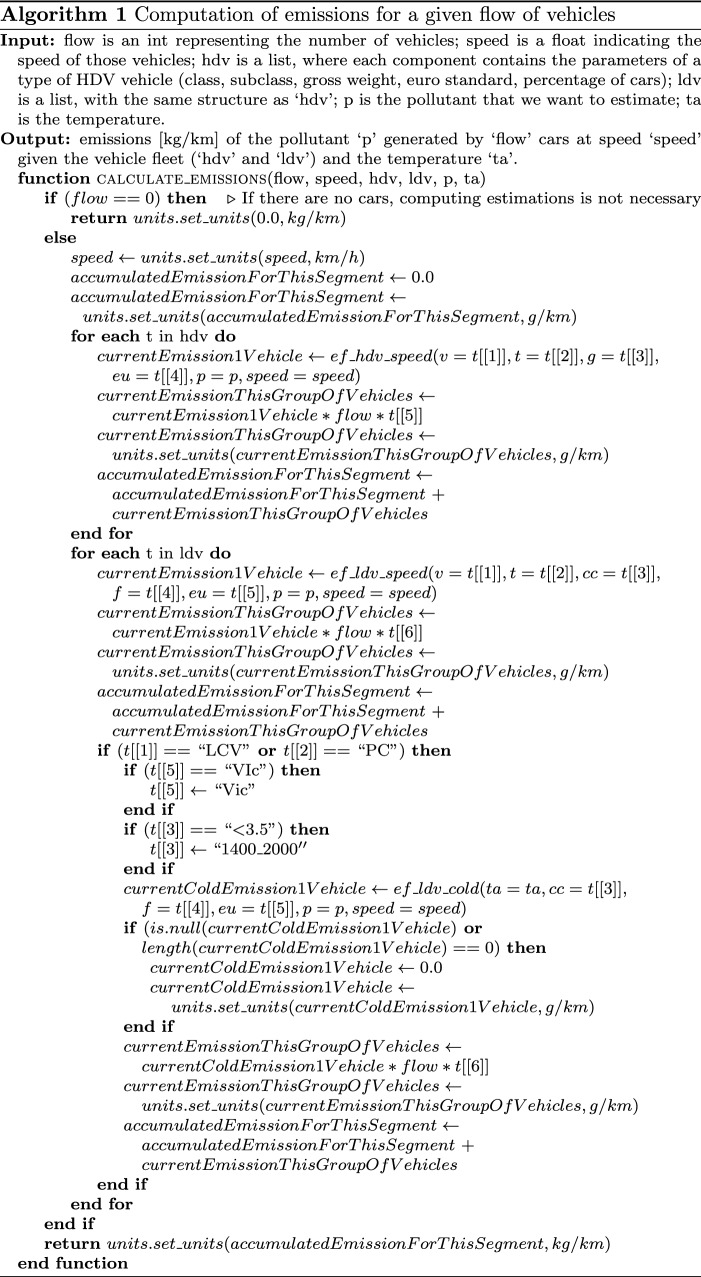



To apply VEIN on the data stored in our database, we had to perform a number of conversions between the historical vehicle fleet data available in our database and the types of parameters required by VEIN (see Tables 2, 3, 4 and 5). In our database, we have a total of 354293 vehicles; about $49.24\%$ of them use diesel as fuel, $50.59\%$ use petrol, and $0.07\%$ use *liquefied petroleum gas (lpg)*.

**Table 2:** VEIN: conversions for the *v* parameter

Class (database)	v (VEIN)
passenger_car	PC
Motorcycle	Motorcycle
Moped	Moped
light_commercial_vehicle	LCV
bus	Coach /Ubus
heavy_duty_truck	Trucks

**Table 3:** VEIN: conversions for the *f* parameter

Fuel (database)	f (VEIN)
lpg	LPG
diesel	D
petrol	G
cng	N/A

**Table 4:** VEIN: conversions for the *cc* parameter

engine_size (database)	cc (VEIN)
0.05	$<{}=50$
0.125	$>{}=50$
0.25	$<{}=250$
0.5	$250\_750$
0.75	$250\_750$
1	$>{}=750$
1.2	$<{}=1400$
1.6	$>{}1400$
2	$1400\_2000$
2.5	$>{}2000$

**Table 5:** VEIN: conversions for the *eu* parameter

emission_standard (database)	**eu (VEIN)**
conventional	PRE
ece 15/04	PRE
euro 1	I
euro 2	II
euro 3	III
euro 4	IV
euro 5	V
euro 6	VI
euro 6 up to 2016	VIc
euro 6 up to 2017	VIc
euro 6 2017-2019	VIc

For performance purposes, values of emissions for a number of combinations of type of car, temperature (month), and speed, can be precomputed. In this way, when the value of the emissions for a given combination of values is needed, the already-precomputed value can be reused.

### Dispersion Model

Once we have estimated the traffic flows in the city (“Traffic Modelling Approach” section) and the pollutants released by those vehicles in each road segment of the city (“Emissions Model” section), we need to estimate how the pollutants will move and disperse in the atmosphere. This depends on factors like the weather conditions (e.g., the presence of wind is a clear factor affecting the movements of pollutants) and the shape of the city (shapes of buildings and their distribution). The goal is to provide a forecast, for the next 48 h, of the air quality (i.e., to supply an estimation of the concentration of pollutants) within the spatial domain considered.

#### Graz Lagrangian Model (GRAL)

For the estimation of atmospheric pollutant dispersion, we use a Lagrangian particle dispersion model. Specifically, we use the Graz Lagrangian Model, GRAL [25], which combines a Lagrangian model and a microscale flow field model for predicting particles dispersion in the urban atmosphere. As a result of the simulations, values of the concentration of the different pollutants considered are obtained.

As described in the documentation [45], the key idea behind Lagrangian models is the tracing of many fictitious particles moving on trajectories within a 3D wind field. The microscale wind field model is used to compute the flow around obstacles. It is based on the Reynolds-average Navier-Stokes equations and uses a standard k-$\varepsilon$ turbulence model [44]. GRAL provides two alternatives for the computation of pollutant concentration time series:

- *Steady-state mode (standard)*. In this case, particles are tracked until they leave the model domain independently of the time they need to do so.
- *Transient mode*. In this case, particles are only tracked until a predefined dispersion time is elapsed. Moreover, the 3D concentration fields obtained considering the previous weather situation (simulation for the previous hour) are stored and reused as starting point for the following weather situation (simulation for the next hour).

The input of pollutant emissions is specified in GRAL by defining different *source groups*. Each source group corresponds to an aggregation of pollutant emissions that follow the same pattern and can be modulated during the different hours of the day, indicating peak hours with high emissions and off-peak hours with low emissions. Moreover, several types of emissions can be distinguished in GRAL. For example, emissions due to road traffic are defined as *line sources*, emissions in delimited city areas (e.g., domestic heating emissions) are *area sources*, and emissions that are produced at a specific location (e.g., in an industrial plant) are denoted as *point sources*.

In each simulation (that is, for each hour), GRAL first computes the wind flow field represented by the meteorological conditions specified in the corresponding input file and stores the result in the so-called *gff file*. Subsequently, and considering this wind field, the dispersion of the corresponding pollutant emissions, also specified in the input files, is calculated.

To be able to provide a daily forecast of the atmospheric pollutant dispersion in the city of Zaragoza for the next 48 h, a speed-up strategy has been adopted: a library of gff files has been precomputed for the most frequent weather conditions, which avoids the need to recompute these files, which represents approximately 2/3 of the total GRAL execution time.

#### GRAL Simulation Settings

**Fig. 14:**
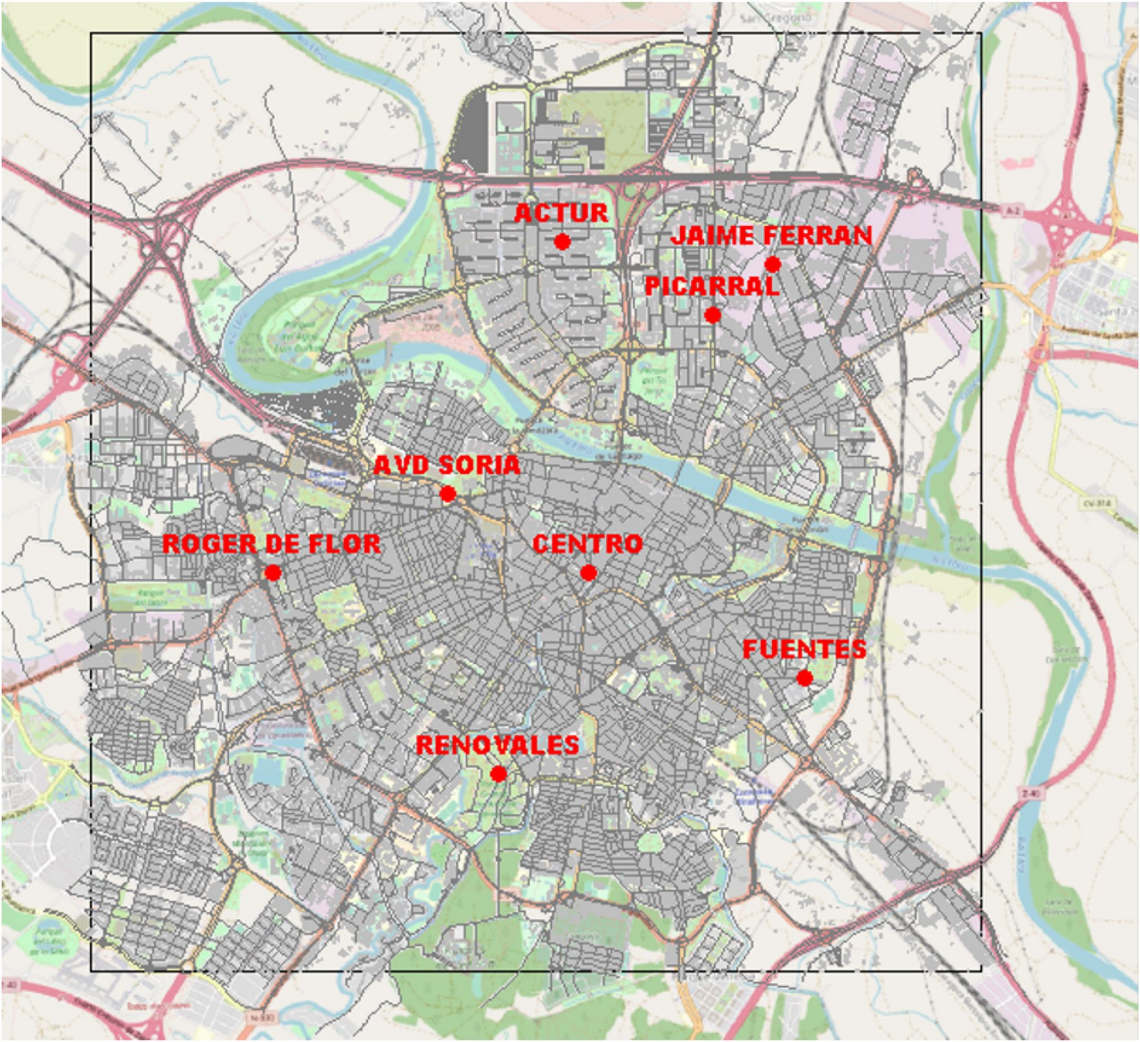
GRAL model domain in the city of Zaragoza and position of the different air quality monitoring stations

In this section, some specifications of our GRAL simulation set-up are provided. We consider a *spatial domain* of 8 km $\times$ 8 km (see Fig. 14), which covers most of the urban area of Zaragoza. For the calculations, we have considered a horizontal grid of 4 m (square cells), thus dividing the whole simulation domain into about 690,000 cells. The smaller the cell, the higher the accuracy, but the computation demands also increase; therefore, a compromise has to be made.

In our case, the simulations are performed in *transient mode*, since it is possible to start considering the previous weather situation (i.e., the one corresponding to the previous hour of the day) and the emissions can be modulated for each weather situation. A predefined simulation time step of 3600 s has been set.

Besides, to cover the whole domain considered for the calculations, 5000 particles are released per second, which according to GRAL developers seems to be enough. The input data required to perform simulations mainly includes meteorological information (“Meteorological Data” section), sufficient data to build a 3D representation of the buildings of the city (“City Building Data” section), and pollutant emission details from urban traffic and other sources such as domestic heating or industrial processes (“Additional Non-traffic Emissions” section).

**Fig. 15:**
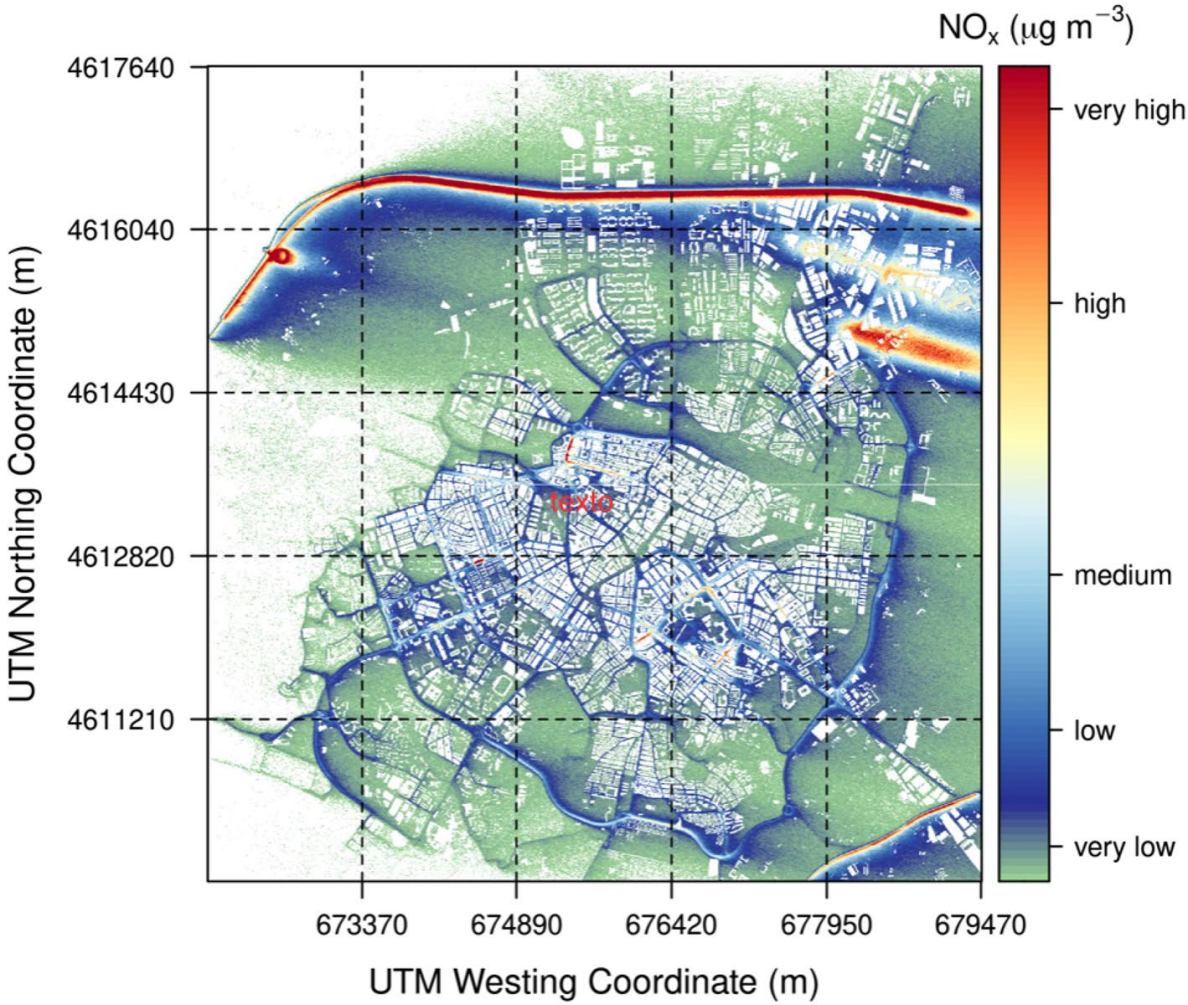
Output of GRAL for July 19, 2020 at 07:00 UTC

For each simulation day, from the output of SUMO, the rush hour (i.e., the hour with the highest number of running vehicles) is identified and VEIN is applied to calculate pollutant emissions. For this hour, the active streets are selected and used as line emissions for the corresponding day. Traffic modulation for the rest of the day is estimated with respect to this rush hour. Moreover, traffic emissions are subdivided into different source groups according to the SUMO traffic cluster analysis and included within GRAL simulations as line sources; the number of source groups changes according to the day of the week and the month of the year. After computation, GRAL returns as output a map of the city indicating the $\hbox {NO}_x$ concentration in all of the cells defined (as an example, see Fig. 15).

GRAL is a complex software that requires significant resources for the spatial domain considered for the city of Zaragoza. In our production environment (see the “Hardware Resources” section), it requires 10–12 h to perform a 24-h forecast of the dispersion of pollutants. Two individual calculations are released every day: a forecast for the next 24 h and a forecast for the following 24 h (thus globally covering a forecast window of 48 h).

## Experimental Evaluation

In this section, we present the experimental evaluation that we have performed to assess our approach. Firstly, in the “Hardware Resources” section, we briefly describe the hardware resources that we have used. Secondly, in the “ Experiments with the Traffic Model” section, we evaluate the traffic model. Finally, in the “Experiments with the Pollution Modelling Approach” section, we focus on the pollution model.

### Hardware Resources

For our experiments, and also to develop an infrastructure that supports the prediction of pollutants on a continuous basis, we have mainly used the following hardware resources:

- The I3A-UZ HERMES supercomputing cluster is the Scientific Linux 5.5-based infrastructure provided by the Aragón Research Institute (I3A) of the University of Zaragoza (UZ). I3A-UZ HERMES, or just HERMES, has more than 1500 parallel processing cores, 6.5 TB of RAM and 175 TB of Luster-based storage, all connected by a 10 Gbps backbone network (second level of connectivity). The queue system used in HERMES is CONDOR.
  For the continuous estimation of pollutants, a machine of 256 GB of RAM and 32 cores with Scientific Linux 5.5 was initially used. In our final production environment, to improve the performance of GRAL, we finally use two different nodes available in HERMES, with the following common configuration: Ubuntu 18.04.4 LTS (GNU/Linux 4.15.0-88-generic x86_64), 64GB RAM, 22 cores, and 1TB of disk capacity. More information about the I3A-UZ-HERMES Cluster is available at https://i3a.unizar.es/en/laboratory/hermes-high-performance-computing-cluster.
- A server computer called Atila, with a CPU Intel Xeon Bronze 3204 CPU @ 1.90 GHz, 6 cores, and 192 GB RAM; it includes different useful support services in the context of our work and other components used to estimate traffic and emissions in a Linux virtual machine within VirtualBox, as well as the database server that stores all the input data.

Besides, other machines have been used for testing and also to hold a previous version of the database.

### Experiments with the Traffic Model

In this section, we perform some experiments to evaluate our traffic modelling approach. First, in the “Evaluation Metrics for SUMO” section, we explain the metrics that we use for the evaluation of the modelling approach. Then, in the “Microscopic Versus Mesoscopic Simulations” section, we compare microscopic and mesoscopic simulations in SUMO. In the “Analysis of Typical Traffic Simulated” section, we analyze typical traffic patterns simulated based on historical data. Finally, in the “Comparison of Traffic Results with Real Values” section, we compare simulated values with real ones.

#### Evaluation Metrics for SUMO

**Fig. 16:**
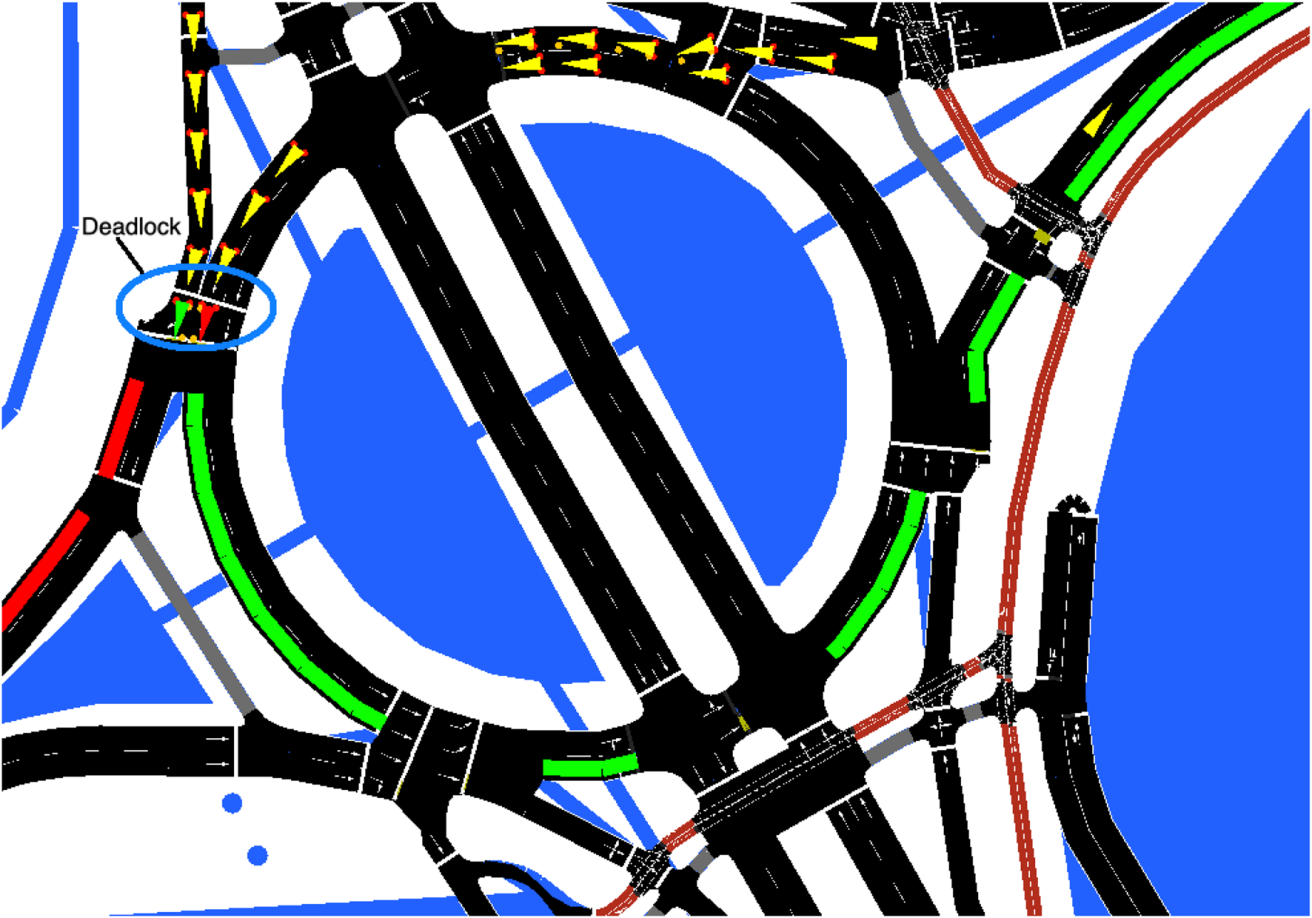
Deadlock between two vehicles at the entrance of a roundabout in Zaragoza during a SUMO simulation; figure extracted from [35]

Two main evaluation metrics have been considered:

- The *simulation error*. As commented in the “Traffic Modelling Approach” section, we have some real traffic data measured/expected in some specific streets of the city and our traffic model must estimate the traffic in all the streets of the city by simulating the flow of vehicles along the roads. Therefore, a key evaluation metric to consider is the *absolute hourly simulation error*, which is the difference between the real traffic measured in the streets that are being monitored (i.e., covered by one of the 46 static traffic devices) and the traffic generated in the simulation with SUMO, for each hour. As an example, if a static traffic device has measured 700 cars in an hour but in the simulation only 620 cars pass by that station, then the simulation error for that hour and location is 80 cars.
  Ideally, the simulation error should be 0. However, as the amount of real observations is small, we have to artificially generate realistic trajectories throughout the whole city based on the real observations, which will lead to some errors in the streets where the traffic is being monitored.
  A simulation error of *n* vehicles could be considered large, medium, or small depending on how big this number is in comparison with the real number of vehicles that have been observed. It is therefore convenient to be able to interpret the absolute simulation error in relative terms. Specifically, the *simulation error rate* for a given hour and static traffic device can be computed by dividing the absolute simulation error for that device and hour by the real observation (i.e., the real traffic at that station and time).
- The *number of teleports*. SUMO avoids potential simulation deadlocks and undesirable situations by automatically teleporting vehicles that have been waiting (without moving) for a while in front of an intersection (by default, 5 min) or that suffer a collision [20]. As an example, a deadlock between two vehicles is shown on the left part of Fig. 16 (the vehicles are represented as triangles in the GUI of SUMO): the green vehicle wants to enter the roundabout and the red vehicle wants to exit it, but each vehicle waits for the other one to move to avoid a potential collision, which leads to a deadlock that will only be solved by teleporting one of the vehicles. We can consider teleports as a simulation hack used to guarantee that the simulation will keep progressing in a suitable way, but obviously automatic displacements of vehicles along the roads are not desirable, even though SUMO considers the average speed of the edges when performing the teleporting and the vehicle is reinserted into the network as soon as this becomes possible (i.e., when there is enough space on the target lane). Therefore, the number of teleports should be as small as possible.
  It should be noted that many of those deadlocks can be solved by manually editing the road network (file .net.xml) by using the graphical network editing tool *netedit* [22] provided by SUMO (e.g., to change the priority of the lanes, add lanes, etc.). However, this leads to a solution which is prone to errors (the real layout must not be changed, even if that change avoids the deadlocks), time-consuming (each problematic point must be carefully edited by a human), and difficult to maintain (as the process cannot be automated, if we download an updated roadmap then all the changes have to be re-applied manually over the new up-to-date map).

To compute the simulation error, we need real data to compare with the simulated data. As described in the “Components Developed for Traffic Modelling” section, a traffic predictor component is in charge of predicting the expected traffic flow that will be measured by the traffic stations on a (future) date for which no actual data is (yet) available, in case this is necessary (i.e., if we want to simulate traffic for future dates, rather than for past dates for which real historical data is available). Based on historical data corresponding to the dates between January 1, 2018 and March 24, 2019, the linear traffic prediction model described in the “Components Developed for Traffic Modelling” section leads to an adjusted $R^2$ of 0.7736 (more than $75\%$ of the variance is explained by the model).

Concerning teleports, through experimentation, we have observed that the number of teleports is particularly high in the case of microscopic simulations. We have also observed that the trends regarding the number of teleports vary along the day: as expected, peak hours (when the number of vehicles circulating is high) lead to higher numbers of deadlocks and therefore to more teleports. The likelihood of teleports can be reduced by manually editing the maps through a trial-and-error procedure: when a simulation bottleneck is observed, causing teleports, we can try to fix it by editing the map. For example, by manually editing 71 intersections in the city of Zaragoza, we could reduce the number of teleports in a typical day from a total of 1860 to 81. However, as commented before, a manual editing of the map has several disadvantages.

#### Microscopic Versus Mesoscopic Simulations

**Fig. 17:**
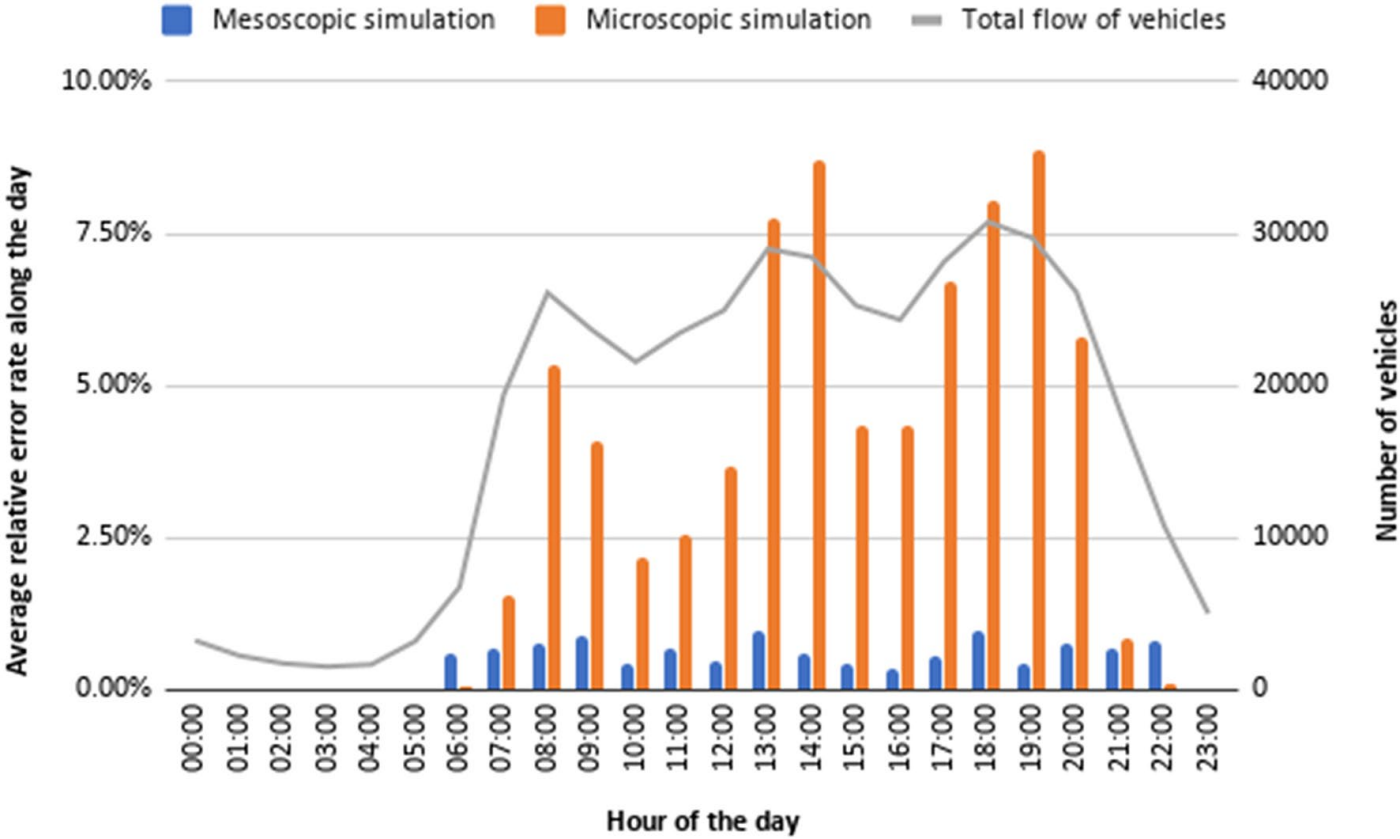
Hourly traffic simulation errors along a day; figure extracted from [35]

In this section, we compare mesoscopic simulations with microscopic simulations and notice that mesoscopic simulations lead to a smaller number of errors in terms of the final traffic flows obtained when compared with the expected traffic flows at the locations with traffic monitoring stations. As an example, the maximum hourly error (maximum value of the differences between the expected traffic flows and the simulated traffic flows during each hour of the day at the edges with monitoring stations) for the 21st of June, 2020, was 300 with the mesoscopic simulation and 2638.4 with the microscopic simulation; the corresponding average relative error rate (average values of those differences computed as percentages over the expected traffic flow at each edge) was $1.34\%$ with the mesoscopic simulation and $10.55\%$ with the microscopic simulation. The simulation errors along that day can be seen in Fig. 17, which shows how the simulation error rates increase with the number of vehicles (i.e., during the peak hours) when a microscopic model is used; however, with the mesoscopic model there are variations but the error rate keeps quite stable along the day. Only in the cases of a very low flow of vehicles the microscopic simulation has low errors comparable to those obtained with the mesoscopic simulation (even slightly lower in some cases, like at 6:00 and at 22:00).

**Fig. 18:**
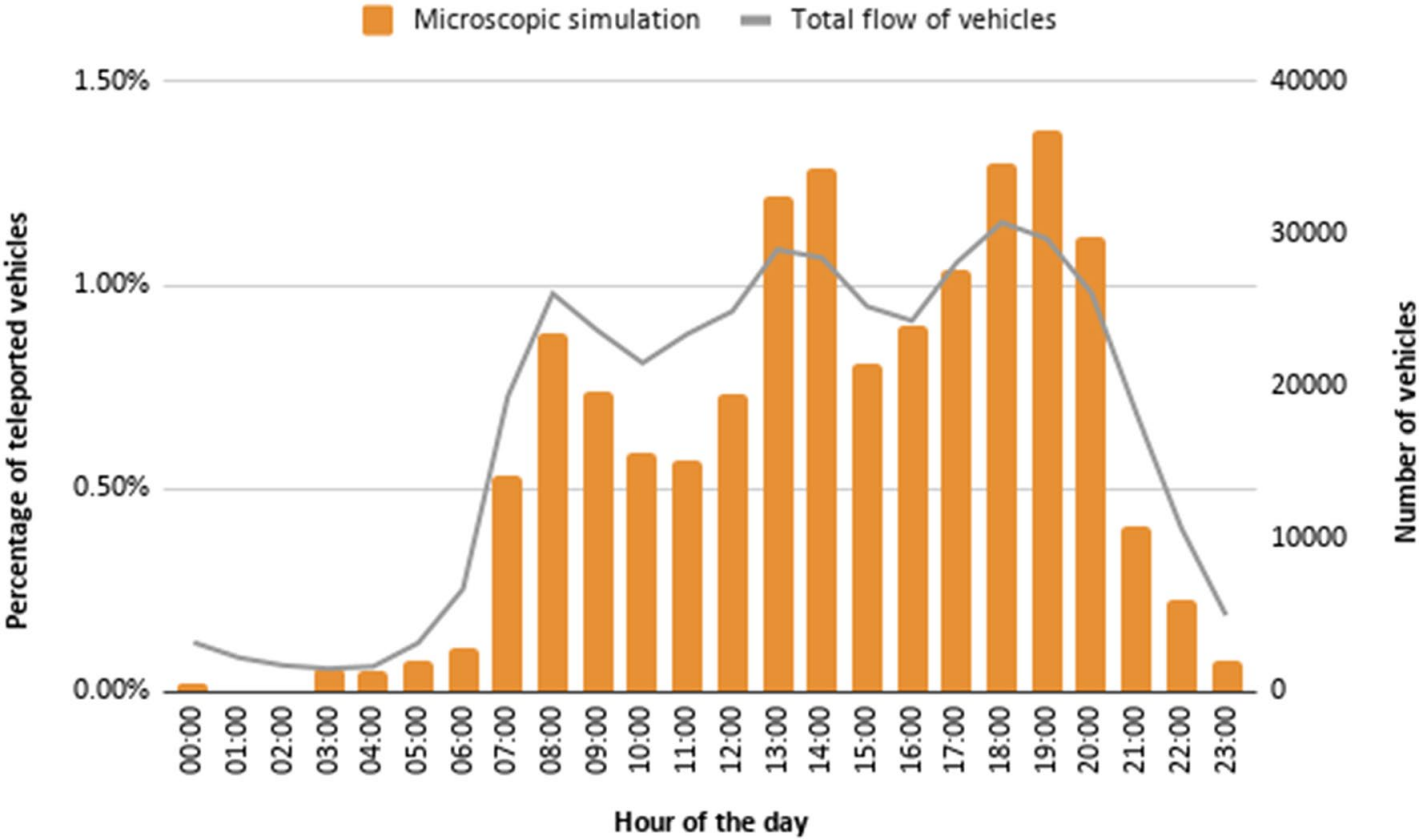
Hourly vehicle teleports along the day with a microscopic traffic simulation; figure extracted from [35]

Regarding the number of teleports, we have also noticed that the percentage of teleported vehicles with a microscopic simulation is considerably higher than with a mesoscopic simulation. Besides, we have observed that the microscopic model is quite more sensitive to small changes in the road layout (e.g., the presence or absence of traffic lights in a roundabout can lead to deadlocks that are solved by SUMO through teleporting). Figure 18 shows the percentage of vehicles teleported along the day when a microscopic simulation is used. Again, we can observe that the number of errors increases with the number of vehicles (i.e., the error is higher during the peak hours).

#### Analysis of Typical Traffic Simulated

**Fig. 19:**
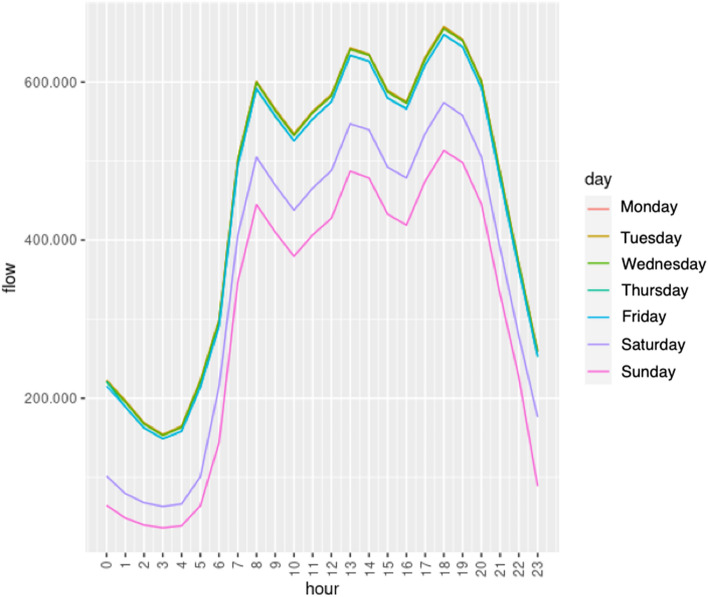
Average hourly traffic flow year

First of all, we show some results concerning SUMO’s daily simulations of a year based on historical traffic data provided by the City Council of Zaragoza. Specifically, our goal is to determine how traffic behaves throughout the day, week and year. In Fig. 19, the average hourly traffic flow (total number of vehicles) for each day is shown. We can notice that the lowest traffic flow is reached at night, between 0 AM and 5 AM. Besides, there are some traffic peaks, at 8 AM, at 1 PM and 6 PM; these are the typical hours of entry and exit from work in the city of Zaragoza. In the figure, it might seem that the lines for some days of the week are missing, but actually what happens is that some lines overlap significantly, specifically in the case of the lines corresponding to Monday, Tuesday, Wednesday and Thursday; the reason is that the traffic flow is very similar on these weekdays.

**Fig. 20:**
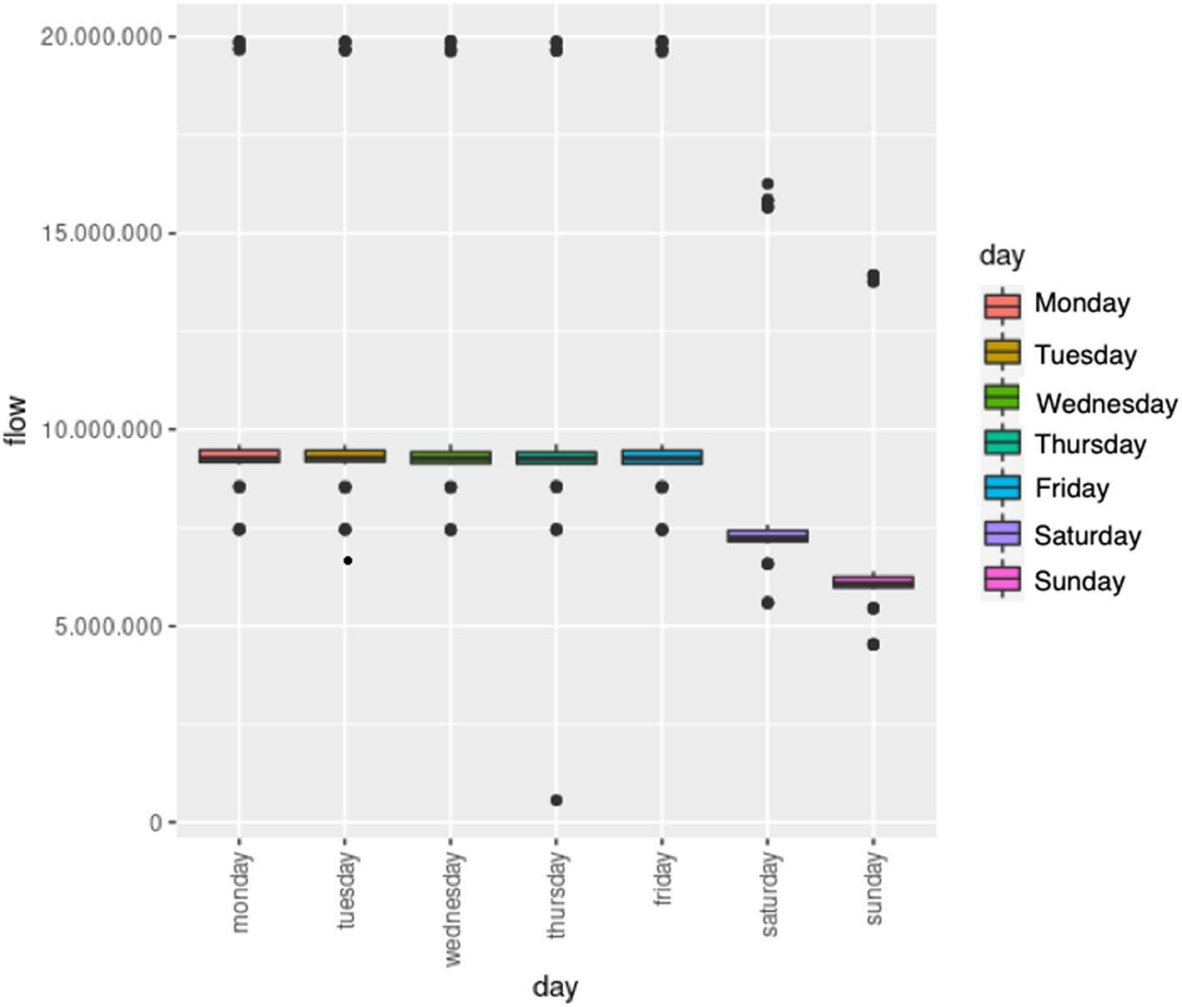
Average daily traffic flow in a week: boxplot

Furthermore, in Fig. 20, we show the average traffic flow for each day of the week. As shown in the figure, the traffic flow is very similar on any weekday, and clearly lower on weekends. The day with less average traffic is Sunday.

**Fig. 21:**
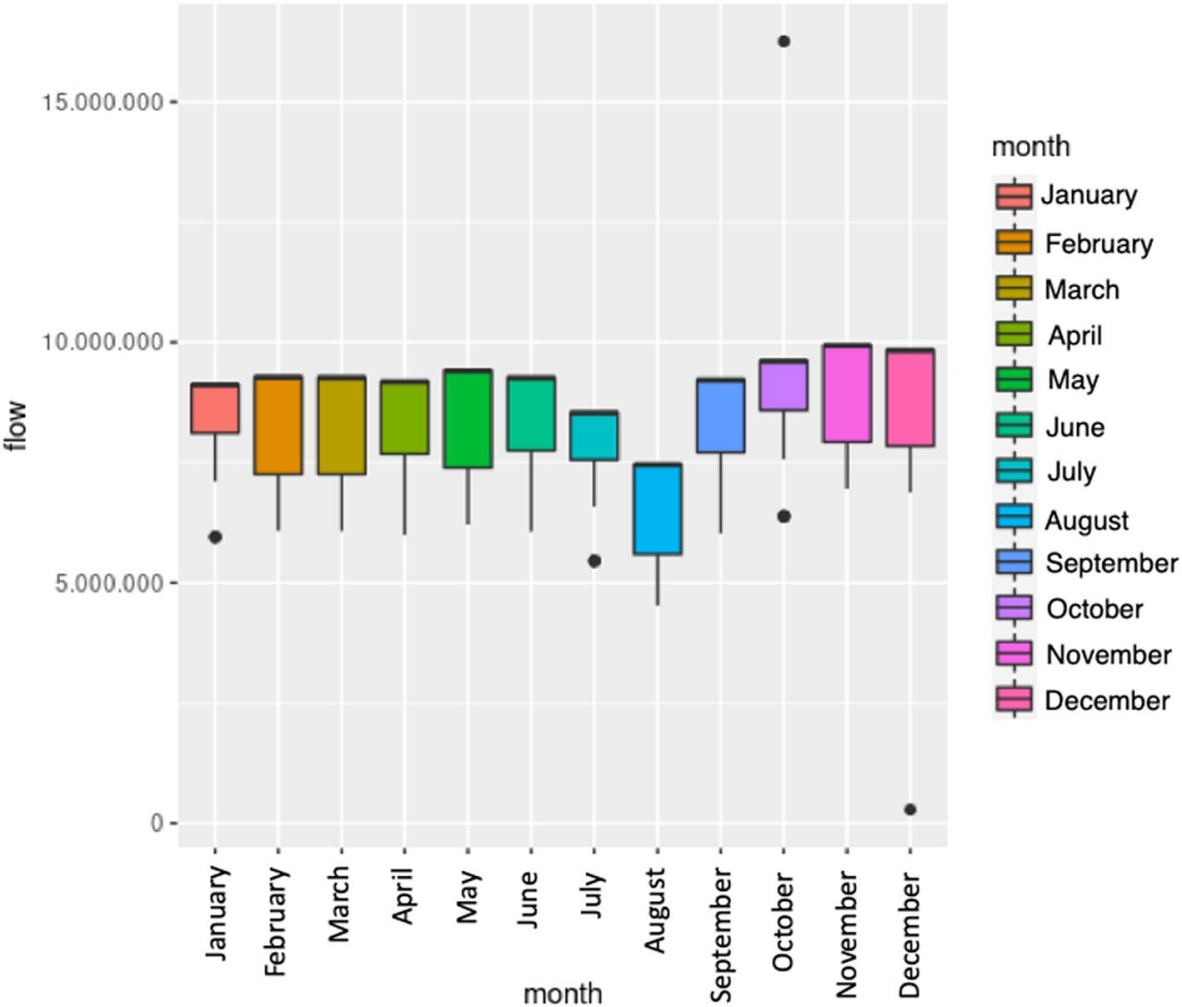
Average daily traffic flow per month: boxplot

**Fig. 22:**
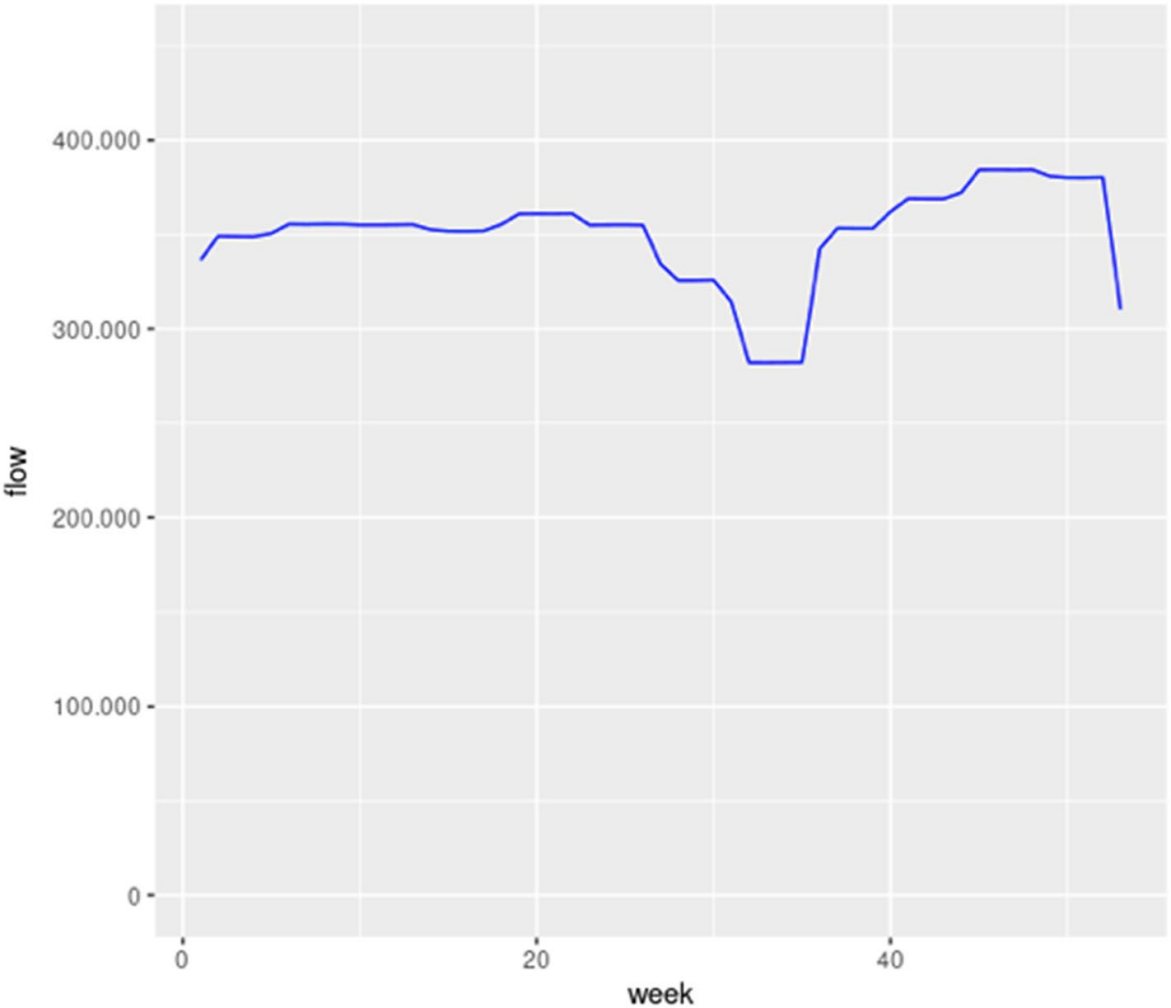
Average hourly traffic flow per week

Figure 21 shows a comparison of the traffic flow in the different months of the year. It can be seen that the average traffic flow is quite similar from January to June. Then, a significant reduction in traffic can be seen in the months of July and August, especially in August. This was expected, as these months are usually the period when many people take their holidays and leave the city for several weeks. After August, the rest of the months until the end of the year (September, October, November, and December) behave like the initial months of the year. In fact, it can be stated that the average traffic flow is almost the same every month, except for July and August. We also show in Fig. 22 the average traffic flow per hour and week of the year, where we can see that the traffic is quite similar along the year except for the weeks that correspond to July and August.

To sum up, several expected results have been observed. Thus, the traffic flow is higher on weekdays. Moreover, the traffic flow is lower at night. There are also some traffic peaks along the day that match with the typical hours of entry and exit from work. Finally, it has been seen that the traffic flow is almost the same in every month, except in the case of July and August due to usual holidays.

#### Comparison of Traffic Results with Real Values

**Fig. 23:**
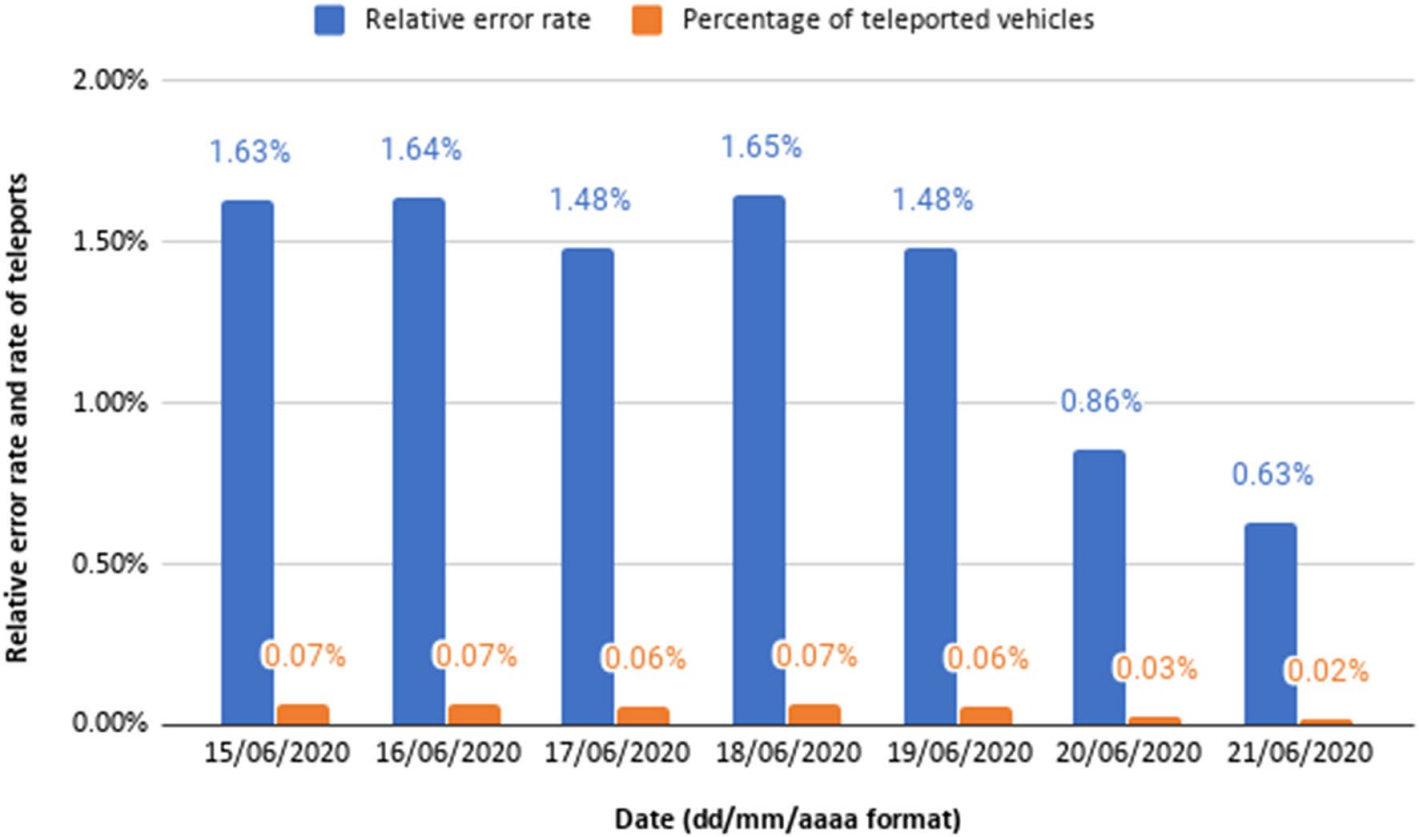
Relative error rate and percentage of teleported vehicles along a week using mesoscopic simulations; figure extracted from [35]

In this section, we evaluate how the traffic modelling approach proposed is able to reproduce real values. As the experimental results presented in the “Microscopic Versus Mesoscopic Simulations” section advise the use of mesoscopic simulations rather than microscopic simulations, we use the former in our current prototype and in the experiments presented in this section. As an example, Fig. 23 shows the relative error rate and the rate of teleports for the simulation of one week of traffic, since Monday (June 15, 2020) until Sunday (June 21, 2020); we can see that the error rates are quite small. Besides, they both decrease significantly during the weekends, which are the periods of less traffic during the week (about $28,58\%$ less traffic for the week simulated). Other experimental results show for example that, when repeating each experiment 10 times, the $95\%$ confidence intervals of the relative error rates are quite small (e.g., $[1.38\%, 1.87\%]$ for Monday and $[0.53\%, 0.72\%]$ for Sunday).

In the following experiments, we simulate a week of 2019 (based on historical data for 2018), from 2019–12–16 (Monday) until 2019–12–22 (Sunday), and compare the simulation results with the real traffic flow data for that week of 2019. More specifically, we focus on the 46 locations where the city council has traffic monitoring stations (see the “Traffic Counts Data” section), as those are the locations where we have real data for comparison. Therefore, we compare the number of vehicles simulated at those points with the real number of vehicles that cross those key points according to the real data available. As explained in the “Evaluation Metrics for SUMO” section, we measure the error as the absolute difference between the traffic flow expected (real data values) and the traffic flow obtained in the simulation (predicted data values), and the relative error is defined as the ratio of the absolute error to the real value (e.g., if the expected traffic flow according to our traffic model at a specific location is 10 vehicles and the error is 15 vehicles, then the relative error would be $50\%$).

**Fig. 24:**
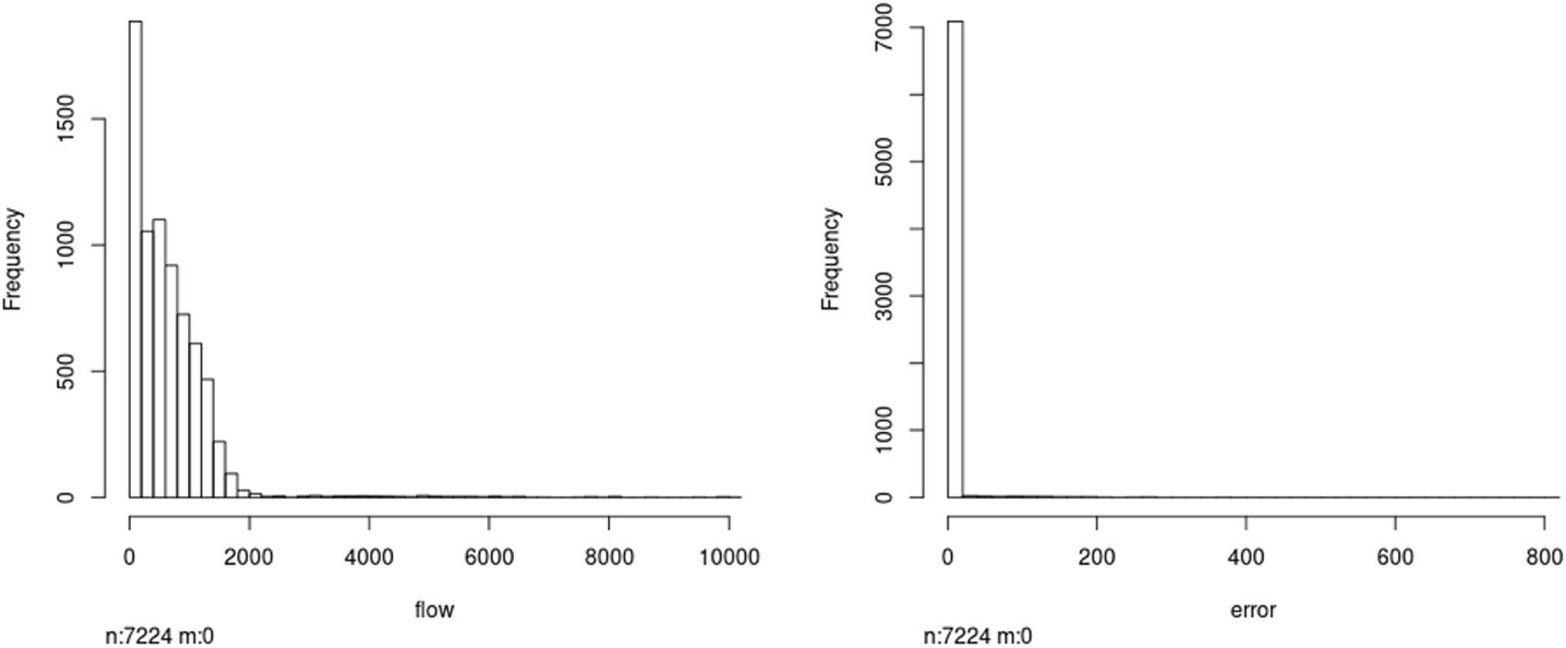
Histogram of traffic flows: real traffic flows (left) and traffic flow errors (right)

Firstly, we have performed an analysis of the traffic flow and the error. The mean traffic flow in 1 h in each of the 46 locations of the city considered (key points, where there is a traffic station) is 643.45 vehicles; the asymptotic confidence interval of this mean (with a significance level of 0.05) is [627.31, 659.60]. On the other hand, the mean error in 1 h in each of the 46 key points of the city is 2.31; the asymptotic confidence interval of this mean is [1.82, 2.81]. Secondly, we consider each key point at each hour during the week and plot the associated histograms: as we can see in Fig. 24, the histograms of both the traffic flow and the traffic flow error are asymmetric. It can be noted that the error is small compared with the expected traffic flow.

**Fig. 25:**
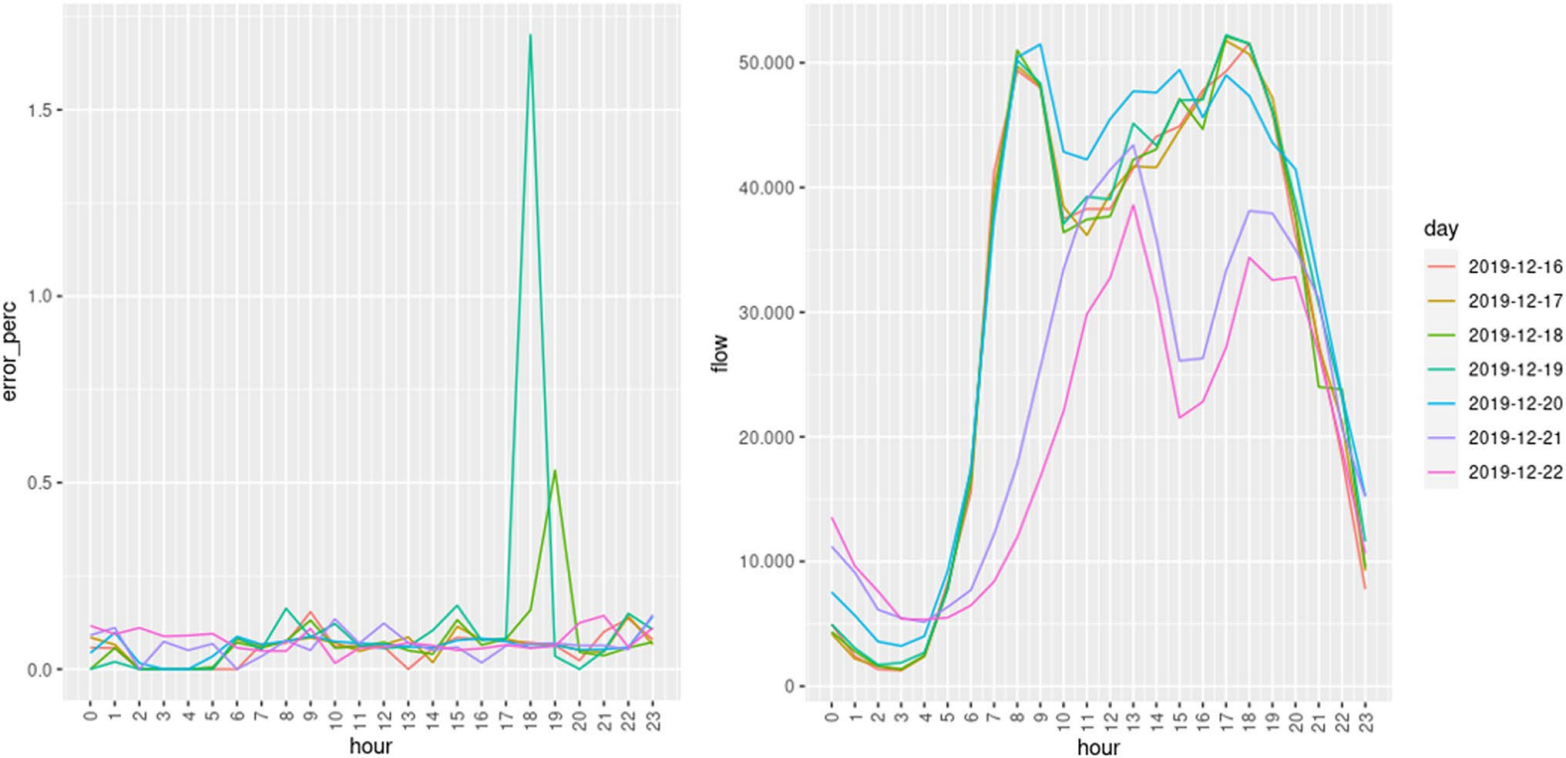
Mean traffic flow per hour and day: relative error (left) and real flow (right)

**Fig. 26:**
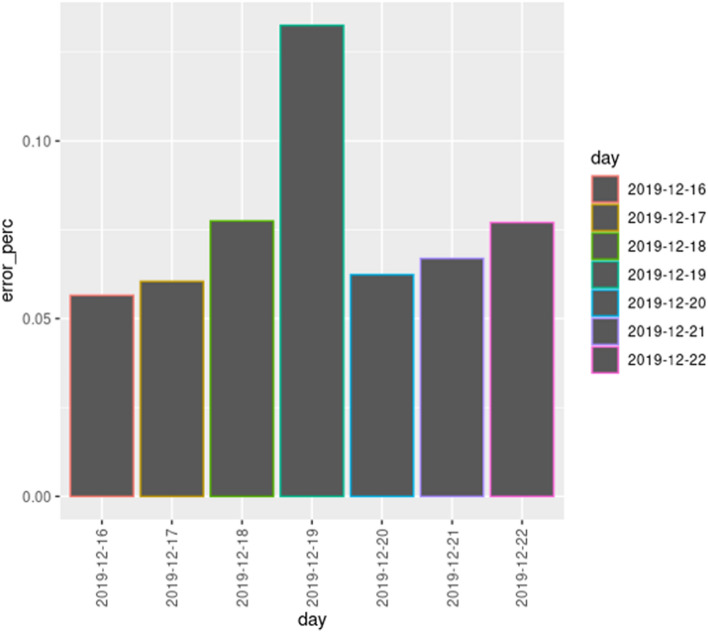
Mean relative traffic flow error per day

On the left of Fig. 25, we show the mean relative traffic flow error per hour for each day of the week (averaged over all the locations), on the right of Fig. 25 we show the real mean traffic flow, and in Fig. 26 we show the corresponding relative traffic flow errors. According to these results, the error rates are very low (never higher that $2\%$) independently of the day and hour, though it can be observed that this error rate is slightly smaller in the hours where the traffic flow is lower. We can observe some peaks on 2019-12-19, but these kinds of peaks are expected, due to the random nature of the traffic model. In conclusion, the traffic flow given by the traffic model is almost identical to the real traffic flow in the 46 key points.

### Experiments with the Pollution Modelling Approach

In this section, we evaluate the pollution modelling approach, described in the “Pollution Modelling Approach” section. More specifically, the modelled $\hbox {NO}_x$ concentrations (i.e., the outputs of GRAL) are compared with real observations at the 8 urban Air Quality Monitoring (AQM) stations described in the “Air Quality Monitoring Stations (AQM)” section, which are located in 8 different location in the city. This evaluation give us information on how far our traffic modelling approach (and the associated emissions) is from the actual pollutant emission values. Similar experiments have been performed to compare the pollution modelling results with the measurements performed by the low-cost quality sensors described in the “Low-Cost Sensors” section, but for brevity we omit the results here because the data provided by the AQM stations are enough to validate the pollution model.

The period considered to perform the comparison is 21 September 2020–1 March 2021. During this period, GRAL simulations have been performed taking into account the whole set of emissions (traffic, domestic heating, industrial combustion and waste management sources) and it corresponds to the period during which the highest pollutant emissions are expected, due to the extra pollution caused by domestic heating during the winter season.

To perform an evaluation of the pollution modelling approach implemented in this work, the European Environment Agency (EEA) recommendations are followed. Specifically, we apply the statistics, graphs and assessment criteria defined in a guidance document [37] elaborated in the context of the Forum for Air quality Modelling in Europe (FAIRMODE) community and the Air Quality Directive (AQD) 2008/50/EC. Therefore, several statistical indicators are computed with the aim of identifying the level of agreement between the results of the model and the observations (real measurements). Further insight can also be given by specific plots, such as the Taylor Diagram. On the other hand, the fulfillment of the standard assessment *Model Quality Objectives (MQO)*, whose goal is the definition of the minimum level of quality to be achieved by a model (to be usable), is analyzed.

**Fig. 27:**
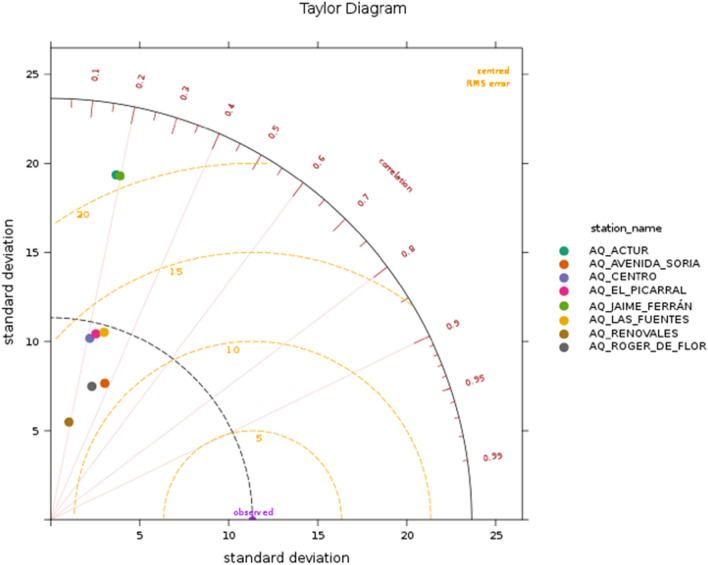
Taylor Diagram displaying the performance of GRAL in reproducing observed concentrations at the eight AQM stations available in the city of Zaragoza for the first day of forecast

**Fig. 28:**
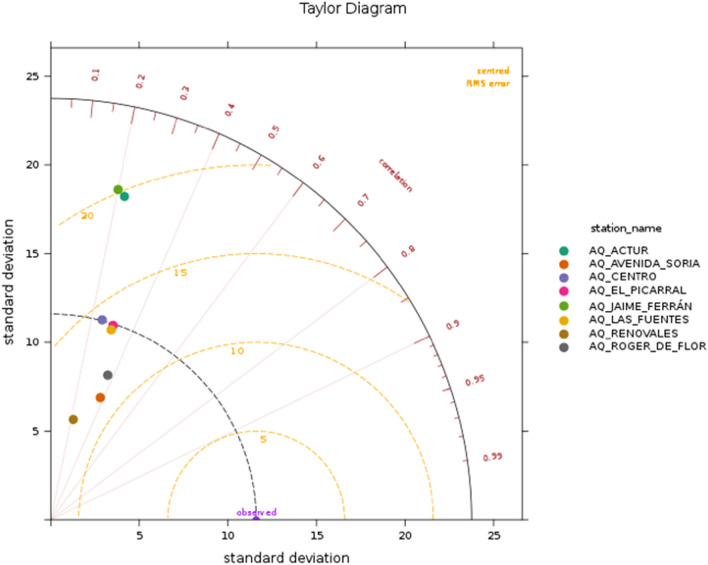
Taylor Diagram displaying the performance of GRAL in reproducing observed concentrations at the eight AQM stations available in the city of Zaragoza for the second day of forecast

In Figs. 27 and 28, the Taylor Diagrams, for the first and the second day of forecast (first 24 h and next 24 h), respectively, are reported. These specific plots enable the visualization of three different statistical indicators in a single diagram: the Pearson correlation coefficient (r), the Centered Root Mean Square Error (CRMSE), and the Standard Deviation of Model (SDM). The results obtained for both days are quite similar; therefore, from now on, for simplicity, we focus only on results for the first day of forecast. The dashed concentric lines originating from the “observed” point show the CRMSE value; therefore, the points which are located the furthest from the “observed” point are the ones with the highest CRMSE values, i.e., where the highest discrepancies can be found. The black dashed line indicates the variability of the measured data; models above that line exhibit greater variability than the actual measurements. Finally, it is also possible to evaluate the linear relationship between the model results and the measurements: points closer to the X-axis will have a higher correlation. According to the figures, the best results are obtained at three AQM stations: *Centro*, *El Picarral* and *Las Fuentes*. There are two AQM where the largest variability can be found: *Actur* and *Jaime Ferrán*; although they are not classified as urban traffic sites, their locations are relatively close to intense traffic streets, including a highway; moreover, Zaragoza is a windy city, which could explain the high variability of the results found for those two AQM.

A quantitative estimation of the agreement between simulated and observed concentrations was also assessed following several statistical metrics proposed for urban dispersion model evaluation. More specifically, the fraction of predicted values within a factor of two of observations (FAC2), Mean Bias (MB), Mean Gross Error (MGE), Normalized Mean Bias (NMB), Normalized Mean Gross Error (NMGE), Root Mean Square Error (RMSE), Pearson correlation coefficient (r), and Index Of Agreement (IOA) were computed using *Openair* [9], which is an open source R package for air quality data analysis. Table 6 summarizes all these metrics for the eight AQM stations, for modelled and observed $\hbox {NO}_x$ concentrations between September 21st 2020 and March 1st 2021, for the first day of forecast. The Mean Bias (MB) is reported as µg/m^3^. Values of the IOA close to 1 represent a better model performance. Negative values of metrics such as MB or NMB indicate that the predicted concentrations are below the real observations. In other words, in general, our model is underestimating the emissions of pollutants. This can be explained by the fact that we are considering only the main sources of pollutants; moreover, in the case of domestic heating and industrial activities, we are working with data that are not up-to-date, more specifically with estimations of the Environmental Agency of the Zaragoza Council for the year 2015, since these are the latest data publicly available (see the “Input Data Sources” section). Moreover, we have considered a spatial square domain of the city without considering inputs from the outside of that domain, which are difficult to be estimated and included. However, despite the variability seen in the data shown in the Taylor Diagrams, there are some promising results. For example, in the AQM *Jaime Ferrán*, $45\%$ of the predicted data are within the interval of 0.5–2 of the observations and, according to the calculated MB, in the same AQM, the predicted values are only 2.59 µg/m^3^ on average below the observed pollutant concentrations. To contextualize this value, a yearly mean concentration of 27 µg/m^3^ can be attributed to the city of Zaragoza (calculated considering data from the official website of the Spanish Ministry for Ecological Transition (https://www.miteco.gob.es/en/).

**Table 6:** Statistical metrics for predicted NO$_x$ hourly concentrations for the first day of forecast

Station	Actur	Avda. Soria	Centro	El Picarral	Jaime Ferrán	Las Fuentes	Renovales	Roger de Flor
Records	2971	2930	3004	2712	2862	2945	2793	2886
FAC2	0.26	0.20	0.13	0.17	0.45	0.12	0.07	0.06
MB	$-$ 3.83	$-$ 13.59	$-$ 18.64	$-$ 16.67	$-$ 2.59	$-$ 20.67	$-$ 17.73	$-$ 27.79
MGE	16.20	14.62	20.22	18.44	15.29	21.92	18.20	28.14
NMB	$-$ 0.21	$-$ 0.67	$-$ 0.74	$-$ 0.68	$-$ 0.12	$-$ 0.74	$-$ 0.82	$-$ 0.85
NMGE	0.88	0.73	0.81	0.75	0.70	0.79	0.84	0.86
RMSE	21.16	18.63	23.73	23.15	20.78	27.12	21.62	33.24
r	0.19	0.37	0.21	0.24	0.20	0.27	0.19	0.30
IOA	0.04	0.25	$-$ 0.03	0.20	0.12	0.17	0.05	0.05

**Fig. 29:**
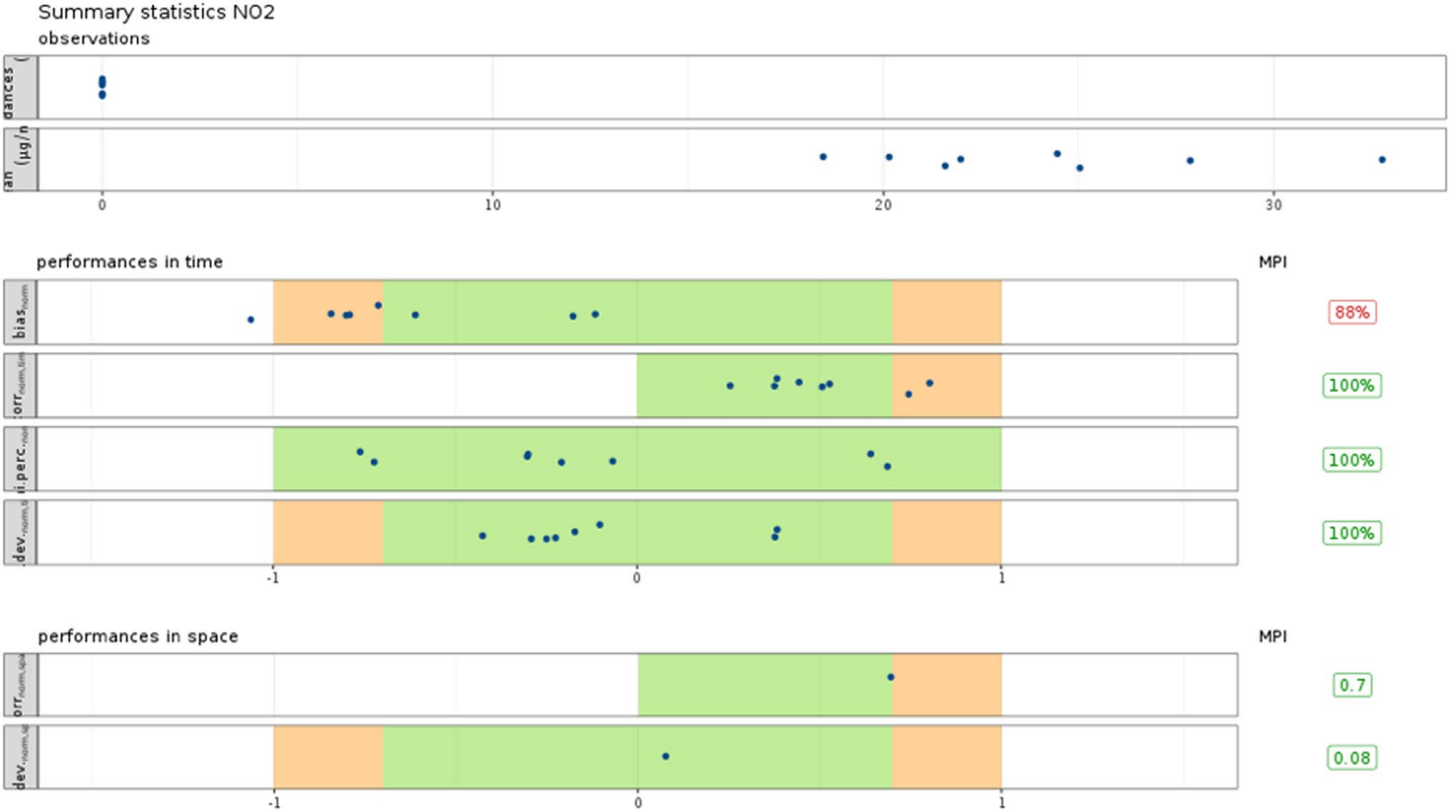
FAIRMODE statistical metrics diagram displaying the performance of GRAL in reproducing observed concentrations at the eight AQM stations in the city of Zaragoza, for the first day of forecast

More statistical metrics for the eight AQM stations are also represented in Fig. 29 for the first day of forecast, including the FAIRMODE [18] validation ranges for those statistical metrics. The first two rows provide the averages of the measurements/observations calculated from the hourly values and the number of times a fixed value of 200 µg/m^3^ is surpassed (air quality violations); 200 µg/m^3^ represents a limit value for $\hbox {NO}_x$ above which air quality is considered not good. For the period considered, no air quality violations have been detected. Rows 3–6 give information about the bias, correlation, ability of the model to capture the highest value, and standard deviation. If the requirements are met, the values fall within the green and orange zone. There is only one AQM that does not meet all the requirements, but only in the case of the first item. Finally, rows 7–8 indicate information of spatial statistics of correlation and standard deviation considering all the AQM stations.

**Fig. 30:**
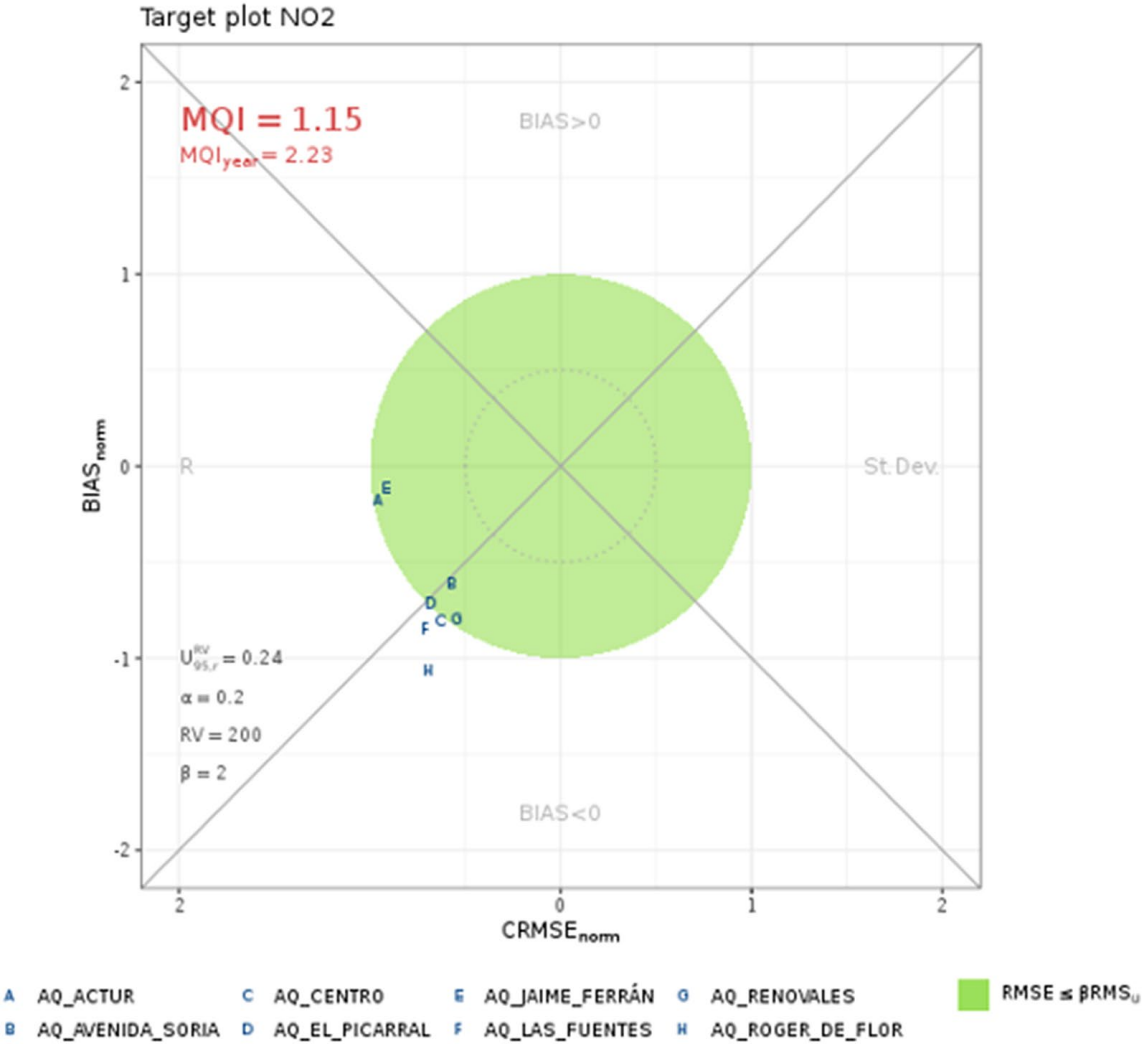
$\hbox {NO}_x$ Target plot for modelled and observed concentrations between September 21st 2020 and March 1st 2021 at the AQM sites, for the first day of forecast

Finally, the fulfillment of the *Model Quality Objectives (MQO)* are analyzed. For this purpose, in the guidance document named previously [37], a *Model Quality Indicator (MQI)* was defined, which is a statistical indicator calculated based on measurements and model results. It is defined as the ratio between the RMSE and a quantity proportional ($\beta$) to the measurement uncertainty (*U95*). The MQO is considered fulfilled when the MQI is not greater than 1. This value can be easily calculated and evaluated by generating the so-called *Target Diagram*, which can be done using Dartle [6], which is an open source R package for air quality model benchmarking. An example of the results obtained for the period between September 21st 2020 and March 1st 2021 at the AQM sites is shown in Fig. 30. In the figure, a green circle indicates the area where the MQO is reached, that is, where the MQI is less than or equal to 1; the smaller circle indicates the region where the MQI is less than or equal to 0.5. The results indicate an average MQI of 1.15, which is close to full compliance with the MQO.

## Conclusions and Future Work

In this paper, we have presented an approach that we have applied in the city of Zaragoza (Spain) for the estimation of pollutants in each area of the city. Our modelling approach is based on the use of three key pieces: (1) the traffic simulator SUMO with real roadmaps of the city and using, as input data, real traffic observations collected by the city’s municipality; (2) the R library VEIN, used to estimate the pollutants produced by the fleet of vehicles; and (3) the particle simulator GRAL, to estimate how the pollutants spread in the atmosphere. Moreover, we have experimentally evaluated our proposal. The results indicate a good accuracy of our traffic model in estimating real traffic flows, whereas the prediction of the dispersion in the atmosphere of the subsequent pollutant emissions is underestimated when compared with real measurements. This is expected since only the main pollutant sources have been considered and some of the data used for the estimations are not up-to-date. Furthermore, the domain selected for calculations has been treated as an isolated square and, in reality, pollution coming from outside that square would actually have also an effect and should be considered to obtain precise results. In any case, the results indicate that the model is close to meet the quality criteria required to be used in practice ($MQI \le 1$) and, despite the variability of the data in some locations, the predictions are not far from real measurements. As a first approach, we can conclude that the results are promising and the methodology applied could be relevant to study other scenarios. The approach followed can help to easily analyze the effect of changes in the vehicle fleet (such as, for example, the impact of decreasing the number of diesel/petrol vehicles in favor of electric ones), or the behavior of vehicles, on urban air pollution.

As future work, we would like to integrate this proposal within a more generic data management framework that tries to encourage citizens to take suitable decisions in face of challenges such as the air pollution. Besides, we could improve the current models by considering additional data sources.

## References

[1] Aiken LS, West SG, Pitts SC, Baraldi AN, Wurpts IC. Multiple linear regression. In: Handbook of Psychology, 2nd ed, chapter 18. American Cancer Society; 2012.

[2] Anastasi G, Antonelli M, Bechini A, Brienza S, D’Andrea E, Guglielmo DD, Ducange P, Lazzerini B, Marcelloni F, Segatori A. In: Urban and social sensing for sustainable mobility in smart cities. 2013 sustainable internet and ICT for sustainability (SustainIT), pp 1–4. IEEE; 2013.

[3] Anenberg SC, Henze DK, Tinney V, Kinney PL, Raich W, Fann N, Malley CS, Roman H, Lamsal L, Duncan B, Martin RV, van Donkelaar A, Brauer M, Doherty R, Jonson JE, Davila Y, Sudo K, Kuylenstierna JC (2018). Estimates of the global burden of ambient PM2.5, ozone, and NO2 on asthma incidence and emergency room visits. Environ Health Perspect.

[4] Ayuntamiento de Zaragoza. Inventario de emisiones 2015. Technical report, Agencia de Medio Ambiente y Sostenibilidad del Ayuntamiento de Zaragoza. 2015. http://www.zaragoza.es/contenidos/medioambiente/agenda21/ECAZ30-inventario-emisiones.pdf. Accessed 2 Mar 2022.

[5] Behera SN, Balasubramanian R. The air quality influences of vehicular traffic emissions. In: Air quality—measurement and modeling. InTech; 2016.

[6] Bonafè G. Dartle: Air quality model benchmarking. A toolkit of R functions for air quality model benchmarking, inspired by the DELTA tool (JRC-IES) and the work of the FAIRMODE WP1. 2017. https://github.com/jobonaf/dartle/. Accessed 2 Mar 2022.

[7] Breiman L (2001). Random forests. Mach Learn.

[8] Camp T, Boleng J, Davies V (2002). A survey of mobility models for ad hoc network research. Wirel Commun Mob Comput.

[9] Carslaw DC, Ropkins K (2012). openair—an R package for air quality data analysis. Environ Model Softw.

[10] Chowdhury D, Santen L, Schadschneider A (2000). Statistical physics of vehicular traffic and some related systems. Phys Rep.

[11] Conticini E, Frediani B, Caro D (2020). Can atmospheric pollution be considered a co-factor in extremely high level of SARS-CoV-2 lethality in Northern Italy?. Environ Pollut.

[12] Curtis L, Rea W, Smith-Willis P, Fenyves E, Pan Y (2006). Adverse health effects of outdoor air pollutants. Environ Int.

[13] Dickson I. Before and after COVID-19: Europe’s traffic congestion mapped. HERE Technologies. 2020. https://360.here.com/covid-19-impact-traffic-congestion. Accessed 2 Mar 2022.

[14] Djahel S, Doolan R, Muntean G-M, Murphy J (2015). A communications-oriented perspective on Traffic Management Systems for smart cities: Challenges and innovative approaches. IEEE Commun Surv Tutor.

[15] Eissfeldt NG. *Vehicle-based modelling of traffic – Theory and application to environmental impact modelling*. PhD thesis, Universität zu Köln. 2004. http://kups.ub.uni-koeln.de/id/eprint/1274. Accessed 2 Mar 2022.

[16] Environmental Protection Agency. Meteorological monitoring guidance for regulatory modeling applications. Technical report, Office of Air and Radiation. Office of Air Quality Planning and Standards. 2020.https://www.epa.gov/sites/default/files/2020-10/documents/mmgrma_0.pdf. Accessed 2 Mar 2022.

[17] Erdmann J. Online-kalibrierung einer mikroskopischen verkehrssimulation. In *ViMOS 2012*. 2012. https://elib.dlr.de/79428. Accessed 2 Mar 2022.

[18] European Commission. FAIRMODE (forum for air quality modelling in Europe). 2007. https://fairmode.jrc.ec.europa.eu/. Accessed 2 Mar 2022.

[19] German Aerospace Center—DLR—SUMO—simulation/meso. 2019 https://sumo.dlr.de/docs/Simulation/Meso.html. Accessed 2 Mar 2022.

[20] German Aerospace Center—DLR—SUMO—simulation/why vehicles are teleporting. 2019.https://sumo.dlr.de/docs/Simulation/Why_Vehicles_are_teleporting.html. Accessed 2 Mar 2022.

[21] German Aerospace Center—DLR—SUMO—NETCONVERT. 2020.https://sumo.dlr.de/docs/NETCONVERT.html. Accessed 2 Mar 2022.

[22] German Aerospace Center—DLR—SUMO—netedit. 2020.https://sumo.dlr.de/docs/NETEDIT.html. Accessed 2 Mar 2022.

[23] German Aerospace Center—DLR—SUMO—simulation/calibrator. 2020.https://sumo.dlr.de/docs/Simulation/Calibrator.html. Accessed 2 Mar 2022.

[24] Google. Google Maps. 2020. http://maps.google.com/. Accessed 2 Mar 2022.

[25] Graz University of Technology. Graz Lagrangian Model (GRAL). 1999. https://gral.tugraz.at/. Accessed 2 Mar 2022.

[26] Han Y, Lam JC, Li VO, Guo P, Zhang Q, Wang A, Crowcroft J, Wang S, Fu J, Gilani Z, Downey J. The effects of outdoor air pollution concentrations and lockdowns on Covid-19 infections in Wuhan and other provincial capitals in China. 10.20944/preprints202003.0364.v1.Accessed 2 Mar 2022. Preprints.org 2020.

[27] Härri J, Filali F, Bonnet C (2009). Mobility models for vehicular ad hoc networks: a survey and taxonomy. IEEE Commun Surv Tutor.

[28] Härri J, Filali F, Bonnet C, Fiore M. VanetMobiSim: Generating realistic mobility patterns for VANETs. In: Third International Workshop on Vehicular Ad Hoc Networks (VANET), pp 96–97, New York. ACM 2006.

[29] Härri J, Fiore M, Filali F, Bonnet C (2011). Vehicular mobility simulation with VanetMobiSim. Simulation.

[30] Hu Y, Barbour W, Samaranayake S, Work D. Impacts of Covid-19 mode shift on road traffic. 2020. arXiv:2005.01610. Accessed 2 Mar 2022.

[31] Ibarra-Espinosa S. VEIN: Vehicular Emissions Inventories. 2018. https://cran.r-project.org/package=vein. Accessed 2 Mar 2022.

[32] Ibarra-Espinosa S. VEINBOOK: Estimating vehicular emissions with the R package VEIN. 2018. https://ibarraespinosa.github.io/VEINBOOK/. Accessed 2 Mar 2022.

[33] Ibarra-Espinosa S, Ynoue R, O’Sullivan S, Pebesma E, Andrade MDF, Osses M (2018). VEIN v0.2.2: An R package for bottom-up vehicular emissions inventories. Geosci Model Dev.

[34] Ilarri S, Delot T, Trillo-Lado R (2015). A data management perspective on vehicular networks. IEEE Commun Surv Tutor.

[35] Ilarri S, Sáez D, Trillo-Lado R. Traffic flow modelling for pollution awareness: The TRAFAIR experience in the city of Zaragoza. In: 16th International Conference on Web Information Systems and Technologies (WEBIST 2020), vol 1, pp 117–128. SCITEPRESS, ISBN 978-989-758-478-7. Online conference due to the COVID-19 pandemic 2020.

[36] INSPIRE Thematic Working Group Buildings. D2.8.III.2 INSPIRE Data Specification on Buildings—Technical Guidelines. 2013. https://inspire.ec.europa.eu/id/document/tg/bu. Accessed 2 Mar 2022.

[37] Janssen S, Guerreiro C, Viaene P, Georgieva E, Thunis P. Guidance document on modelling quality objectives and benchmarking. https://fairmode.jrc.ec.europa.eu/document/fairmode/WG1/Guidance_MQO_Bench_vs2.1.pdf. Accessed 2 Mar 2022. FAIRMODE, Forum for Air Quality Modelling in Europe 2017.

[38] Krauß S. *Microscopic Modeling of Traffic Flow: Investigation of Collision Free Vehicle Dynamics*. PhD thesis, Universität zu Köln. 1998. http://e-archive.informatik.uni-koeln.de/id/eprint/319. Accessed 2 Mar 2022.

[39] Laña I, Ser JD, Padró A, Vélez M, Casanova-Mateo C (2016). The role of local urban traffic and meteorological conditions in air pollution: A data-based case study in Madrid, Spain. Atmos Environ.

[40] Lockhart D, Vaganay M, MacIntyre S, Joseph P (2015). A meta-analysis of the impact of traffic-related air pollution on health and the factors affecting exposure. WIT Transactions on Ecology and the Environment.

[41] Lopez PA, Behrisch M, Bieker-Walz L, Erdmann J, Flötteröd Y, Hilbrich R, Lücken L, Rummel J, Wagner P, Wiessner E. Microscopic traffic simulation using SUMO. In: 21st International Conference on Intelligent Transportation Systems (ITSC); 2018. pp 2575–2582

[42] Mayer H (1999). Air pollution in cities. Atmos Environ.

[43] Ntziachristos L, Samaras Z. EMEP/EEA emission inventory guidebook; road transport: Passenger cars, light commercial trucks, heavy-duty vehicles including buses and motorcycles. Technical report, European Environment Agency. 2016. https://www.eea.europa.eu/publications/emep-eea-guidebook-2016/part-b-sectoral-guidance-chapters/1-energy/1-a-combustion/1-a-3-b-i. Accessed 2 Mar 2022.

[44] Oettl D (2015). Quality assurance of the prognostic, microscale wind-field model gral 14.8 using wind-tunnel data provided by the german vdi guideline 3783–9. J Wind Eng Ind Aerodyn.

[45] Oettl D. Documentation of the Lagrangian particle model GRAL (Graz Lagrangian Model) Vs. 18.1. 2018. 10.13140/RG.2.2.11118.72005. Accessed 2 Mar 2022. Bericht Nr. Lu-01-2018

[46] OpenSim Ltd. OMNeT++. 2000. https://omnetpp.org/. Accessed 2 Mar 2022.

[47] OpenStreetMap Foundation. OpenStreetMap. 2004. https://www.openstreetmap.org. Accessed 2 Mar 2022.

[48] PostGIS Team. PostGIS. 2001.https://postgis.net/. Accessed 2 Mar 2022.

[49] QGIS Development Team. QGIS—a free and open source geographic information system. 2004. https://www.qgis.org/. Accessed 2 Mar 2022.

[50] Samet JM (2007). Traffic, air pollution, and health. Inhal Toxicol.

[51] Sharif A, Li J, Khalil M, Kumar R, Sharif MI, Sharif A. Internet of Things — smart traffic management system for smart cities using Big Data analytics. In *14th International Computer Conference on Wavelet Active Media Technology and Information Processing (ICCWAMTIP 2017)*, pp 281–284. IEEE; 2017.

[52] Sommer C. Veins (Vehicles in Network Simulator). 2006. http://veins.car2x.org/. Accessed 2 Mar 2022.

[53] Sommer C, German R, Dressler F (2011). Bidirectionally coupled network and road traffic simulation for improved IVC analysis. IEEE Trans Mob Comput (TMC).

[54] The PostgreSQL Global Development Group. PostgreSQL. 1996. https://www.postgresql.org/. Accessed 2 Mar 2022.

[55] Trillo R, Ilarri S, Mena E. Comparison and performance evaluation of mobile agent platforms. In: Third International Conference on Autonomic and Autonomous Systems (ICAS). IEEE Computer Society; 2007. pp 41–46

[56] Urra O, Ilarri S. Cognitive Vehicular Networks, chapter 10 “MAVSIM: Testing VANET Applications Based on Mobile Agents”, pp 199–224. CRC Press—Taylor & Francis Group. Print ISBN: 978-1-4987-2191-2, eBook ISBN: 978-1-4987-2192-9 2016.

[57] Worldsensing. Traffic Flow Management in Zaragoza City – Success Story. 2018. https://www.worldsensing.com/wp-content/uploads/2018/01/v1_Plantilla_Success_Story_Bitcarrier_Traffic-Flow-Management-in-Zaragoza-City.pdf. Accessed 2 Mar 2022.

[58] Worldsensing. Bitcarrier—The real-time Traffic Flow Monitoring System. 2020. https://web.archive.org/web/20201001223843/http://www.worldsensing.com/product/bitcarrier/. Accessed 2 Mar 2022.

[59] Wu X, Nethery RC, Sabath BM, Braun D, Dominici F (2020). Exposure to air pollution and COVID-19 mortality in the United States: a nationwide cross-sectional study. Sci Adv.

